# Synthesis of aryl sulfides via radical–radical cross coupling of electron-rich arenes using visible light photoredox catalysis

**DOI:** 10.3762/bjoc.14.228

**Published:** 2018-09-27

**Authors:** Amrita Das, Mitasree Maity, Simon Malcherek, Burkhard König, Julia Rehbein

**Affiliations:** 1 Department of Chemistry and Pharmacy, Institute of Organic Chemistry, University of Regensburg, Universitätsstraße 31, 93053 Regensburg, Germany

**Keywords:** arenes, oxidation, photocatalysis, thiolation, visible light

## Abstract

Electron-rich arenes react with aryl and alkyl disulfides in the presence of catalytic amounts of [Ir(dF(CF_3_)ppy)_2_(dtbpy)]PF_6_ and (NH_4_)_2_S_2_O_8_ under blue light irradiation to yield arylthiols. The reaction proceeds at room temperature and avoids the use of prefunctionalized arenes. Experimental evidence suggests a radical–radical cross coupling mechanism.

## Introduction

**Figure 1:**
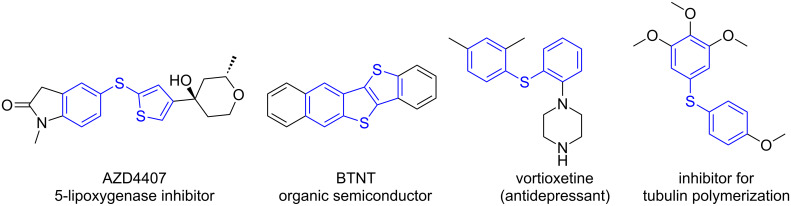
Selected examples of sulfenylated heterocycles used in pharmaceuticals and material chemistry.

**Scheme 1:**
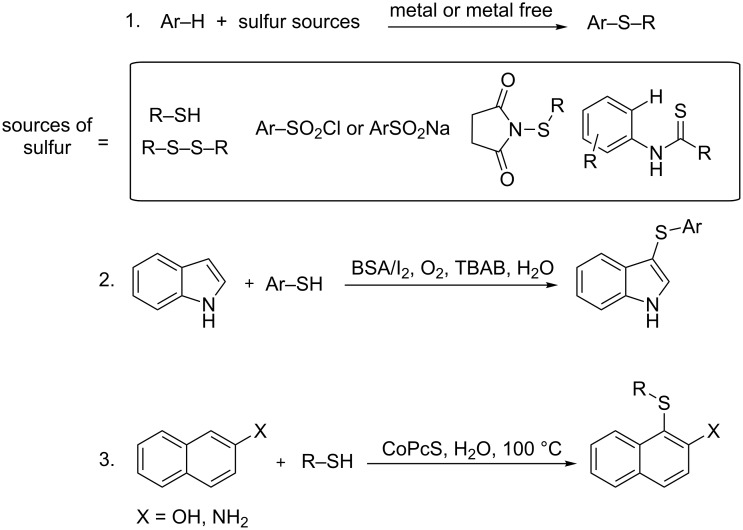
Synthetic routes to organosulfur compounds.

**Scheme 2:**
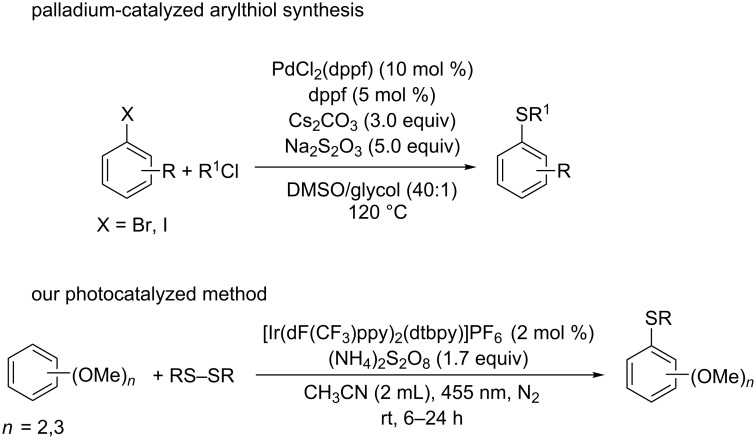
Aryl sulfide synthesis.

The generation of carbon–sulfur bonds is an important task in organic synthesis, because of their abundance in target structures, such as natural products and drugs [1–3]. They are found in organic semiconductors, antidepressant or antileukotriene agents (Figure 1). Three of the five most selling drugs in 2015 were organosulfur compounds. The majority of methods for C–S bond synthesis use transition metal-catalyzed cross coupling of thiols and their derivatives with organohalides [4–6], arylboronic acids [7], aryl triflates [8], and diazonium salts [9]. Typical metals used are palladium [10–13], copper [14–21], nickel [22–24], iron [25–29], cobalt [30–32], and rhodium [33–34]. Aryl sulfides are also synthesized by cross coupling of thiols and aryl Grignard/arylzinc reagents [35–36]. However, most of these methods require harsh reaction conditions, external additives and high temperatures. The reactions need prefunctionalized arenes, while a direct C–S sulfenylation by C–H functionalization would be more desirable and cost effective. So far, only a few reports on direct C–H functionalization using transition metals or metal free [37–39] conditions and different sources of sulfur, for example arylsulfonyl chlorides, sodium arylsulfinates, sulfinic acids and arylsulfonyl hydrazides have been reported (Scheme 1). However, the protocols require prefunctionalized sulfenylating reagents. Recently Lei and co-workers reported a DDQ-mediated selective radical–radical cross coupling between electron-rich arenes and thiols [40]. Miyake et al. reported the visible light-promoted cross-coupling reaction between aryl halides and arylthiols via an intermolecular charge transfer using Cs_2_CO_3_ as base [41]. Two recent reports showed the synthesis of C-3 sulfenylated indoles and 3-sulfenylimidazopyridine via C–H functionalization using Rose Bengal as photocatalyst [42–43]. In general, the arylation reactions use the reductive cycle of the photocatalyst and for this, electron poor arenes are required. In this article, we report the development of a mild and efficient oxidative photocatalytic method of thiolation of electron-rich di- and trimethoxybenzene arenes with aryl disulfides and (NH_4_)_2_S_2_O_8_ as terminal oxidant (Scheme 2).

## Results and Discussion

**Scheme 3:**
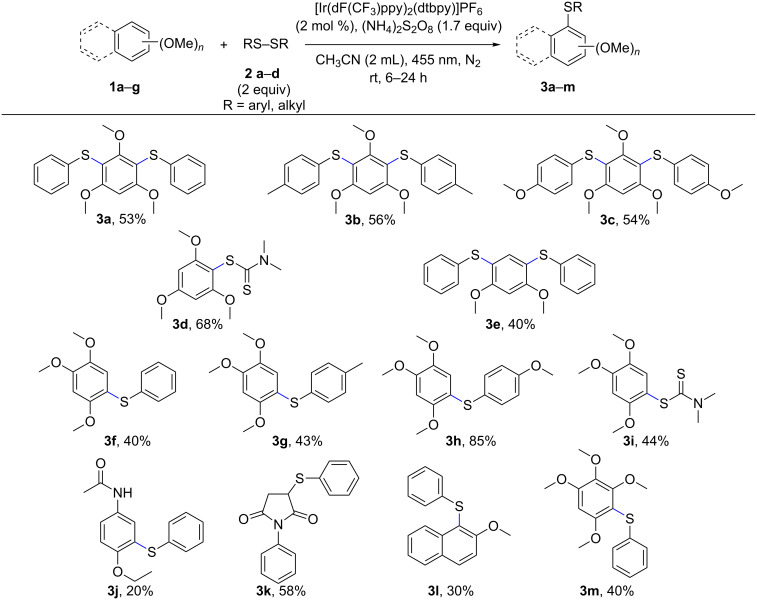
Substrate scope for arylthiol syntheses. The reaction was performed with **1a**–**g** (0.1 mmol) and **2a**–**d** (2 equiv), (NH_4_)_2_S_2_O_8_ (1.7 equiv) and [Ir(dF(CF_3_)ppy)_2_(dtbpy)]PF_6_ (2 mol %) in 2 mL CH_3_CN. For reaction times see Supporting Information File 1.

**Figure 2:**
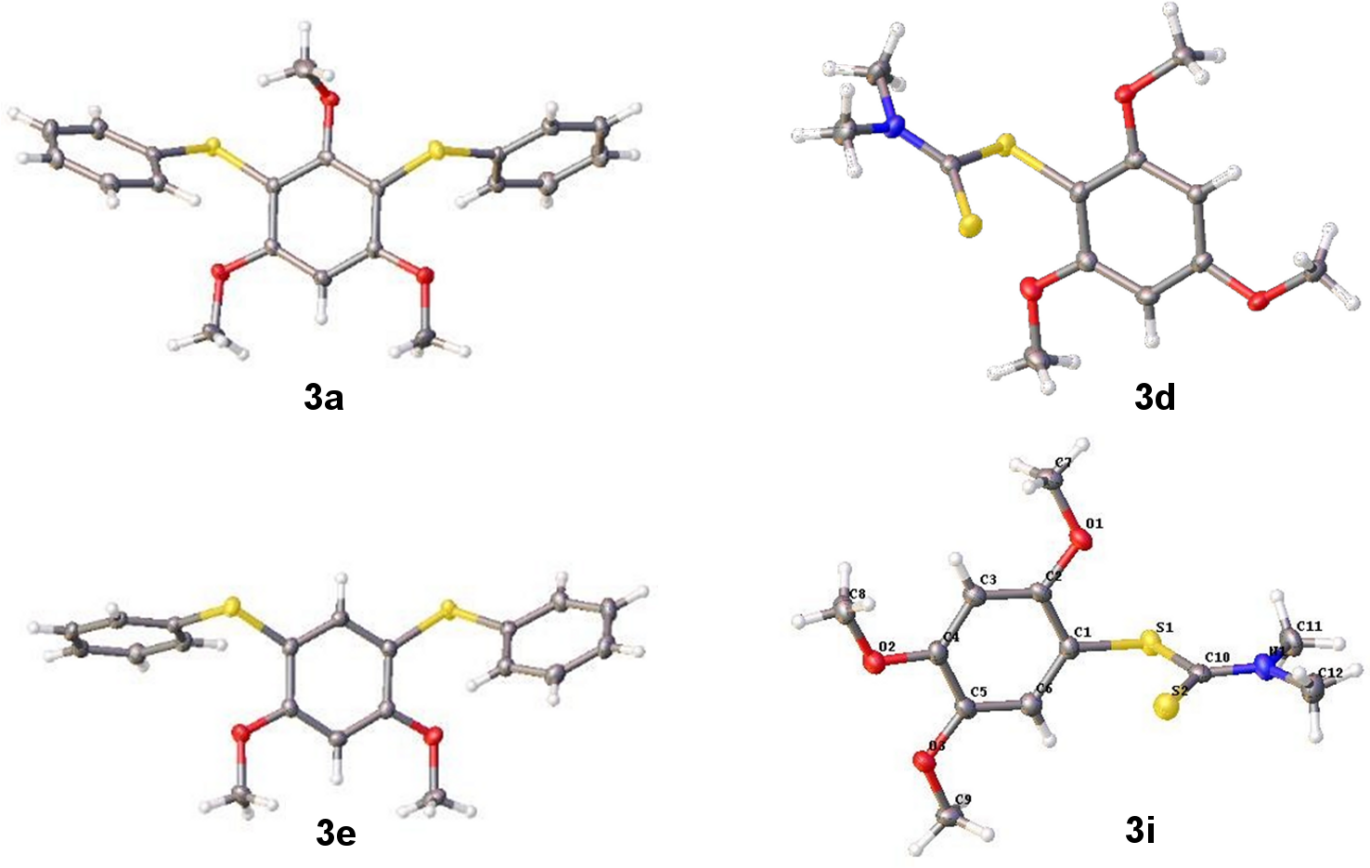
Crystal structures of compounds **3a**, **3d**, **3e** and **3i**.

1,2,4-Trimethoxybenzene and diphenyl disulfide were employed as the model substrates to test our proposal and to optimize the reaction conditions. Our developed photocatalytic method allows the activation of electron-rich alkoxyarenes for the direct C–H sulfenylation reaction using visible light and [Ir(dF(CF_3_)ppy)_2_(dtbpy)]PF_6_ as the photocatalyst. The reaction was carried out under nitrogen under visible-light irradiation at 455 nm. The oxidation potential of this test arene is 1.02 V vs SCE, which allows oxidation by [Ir(dF(CF_3_)ppy)_2_(dtbpy)]PF_6_ having an estimated excited state oxidation potential of 1.21 V vs SCE. Other photocatalysts like Ru(bpy)_3_Cl_2_, Ru(bpz)_3_PF_6_, DDQ, acridinium dyes, Eosin Y, Eosin Y disodium salt and 4-CzIPN were evaluated, but under our reaction conditions either low substrate conversion or the degradation of the photocatalyst was observed (see Supporting Information File 1, Table S1). The organic dye 9-mesityl-10-phenylacridinium tetrafluoroborate completely decomposed in the presence of excess disulfide within 30 minutes of irradiation. [Ir(dF(CF_3_)ppy)_2_(dtbpy)]PF_6_ was found to be the best photocatalyst and in this case, CH_3_CN was the best solvent compared to DMF, DMSO and DCE. When thiophenol was used as the sulfur source, diphenyl disulfide was obtained as a major side product, which in turn hindered the arylation process and resulted in several other oxidized products of thiophenol. So, the readily available diphenyl disulfide was added as the thiolating agent. Addition of excess disulfide (e.g., 5 equivalents) resulted in the formation of thiophenol as a major side product along with other oxidized sulfur species. The amount of disulfide was varied from 0.5 equivalents to five equivalents; 1.7 equivalents of disulfide gave the best result. The photocatalytic reaction was very slow when air was used as an oxidant, also it led to various oxidation products of the sulfur and the degradation of the photocatalyst was observed upon irradiation. Therefore, (NH_4_)_2_S_2_O_8_ was used as terminal oxidant. The addition of *tert*-butyl hydroperoxide (TBHP) as an oxidant led to degradation of the reaction mixture, also when nitrobenzene (PhNO_2_) was used as oxidant, trace amounts of product were observed along with the formation of aniline, likely arising from the regeneration of the catalyst. Control experiments confirmed that light and photocatalyst were essential for the arylation reaction (see Supporting Information File 1, Table S2). With these conditions in hand, electron rich di- and trimethoxyarenes were reacted. The reactions were complete within 6 to 24 hours and the products were obtained in moderate yields. The best yield of 85% was observed when the electron-rich bis(4-methoxyphenyl) disulfide was employed as the thiolating agent (**3h**). With symmetrical arenes, diarylation was observed. The initially formed mono-arylated product is more reactive than the starting material and reacts to the diarylthiol product. When 1-phenyl-1*H*-pyrrole-2,5-dione was employed as the arene, the sulfenylation occurred exclusively at the double bond instead at the arene to give the product **3k** in 58% yield and a trace amount of diarylation product of the aromatic ring. When the more difficult to oxidize 2-methoxynaphthalene was used as substrate, the product **3l** was obtained in only 30% yield indicating the limit of the scope of the method. The substrate scope is shown in Scheme 3. The structures of compounds **3a**, **3d**, **3e** and **3i** in the solid state were determined by X-ray structure analysis (Figure 2).

**Scheme 4:**
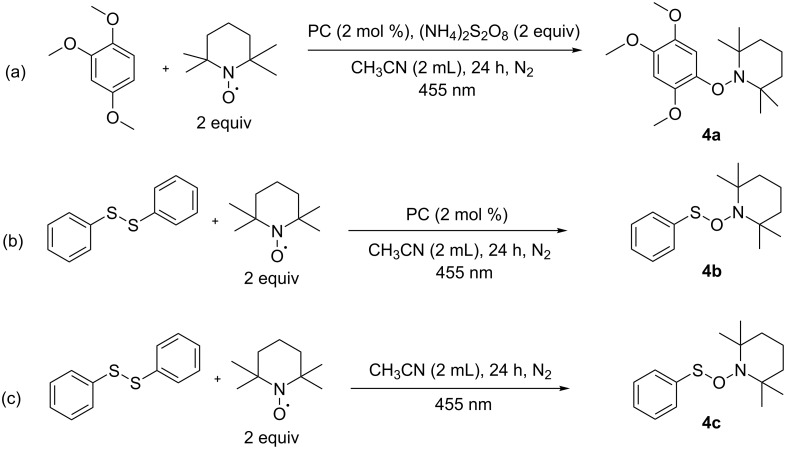
Radical trapping experiments.

We performed various control experiments to support the proposed reaction mechanism, which is shown in Scheme 4. Two equivalents of 2,2,6,6-tetramethylpiperidyl-1-oxyl (TEMPO), a radical scavenger were added to 1,2,4-trimethoxybenzene (Scheme 4a), in the presence of [Ir(dF(CF_3_)ppy)_2_(dtbpy)]PF_6_, ammonium thiosulfate and 455 nm LED irradiation. The reaction mixture was analyzed by mass spectrometry, which showed the molecular ion indicating the formation of the proposed TEMPO adduct with the arene radical intermediate. Also, when diphenyl disulfide was irradiated with TEMPO in the presence and absence of the photocatalyst, (Scheme 4b and Scheme 4c) the adduct 2,2,6,6-tetramethyl-1-((phenylthio)oxy)piperidine was obtained in both cases. See Supporting Information File 1 for the HRMS analysis of the TEMPO adduct. These radical trapping experiments show that initially a radical cation of the arene is formed by the excited photocatalyst, which then is trapped by the radical scavenger TEMPO. S–S bond cleavage has been reported for alkyl and aryl disulfides in an oxidative [44–46] and triplet sensitized mechanism [47]. It is also well known in literature that aromatic disulfides are cleaved homolytically under UV irradiation yielding the corresponding radicals [48]. A recent study from Nicewicz showed that an aryl disulfide could be cleaved by irradiation with visible light [49].

**Figure 3:**
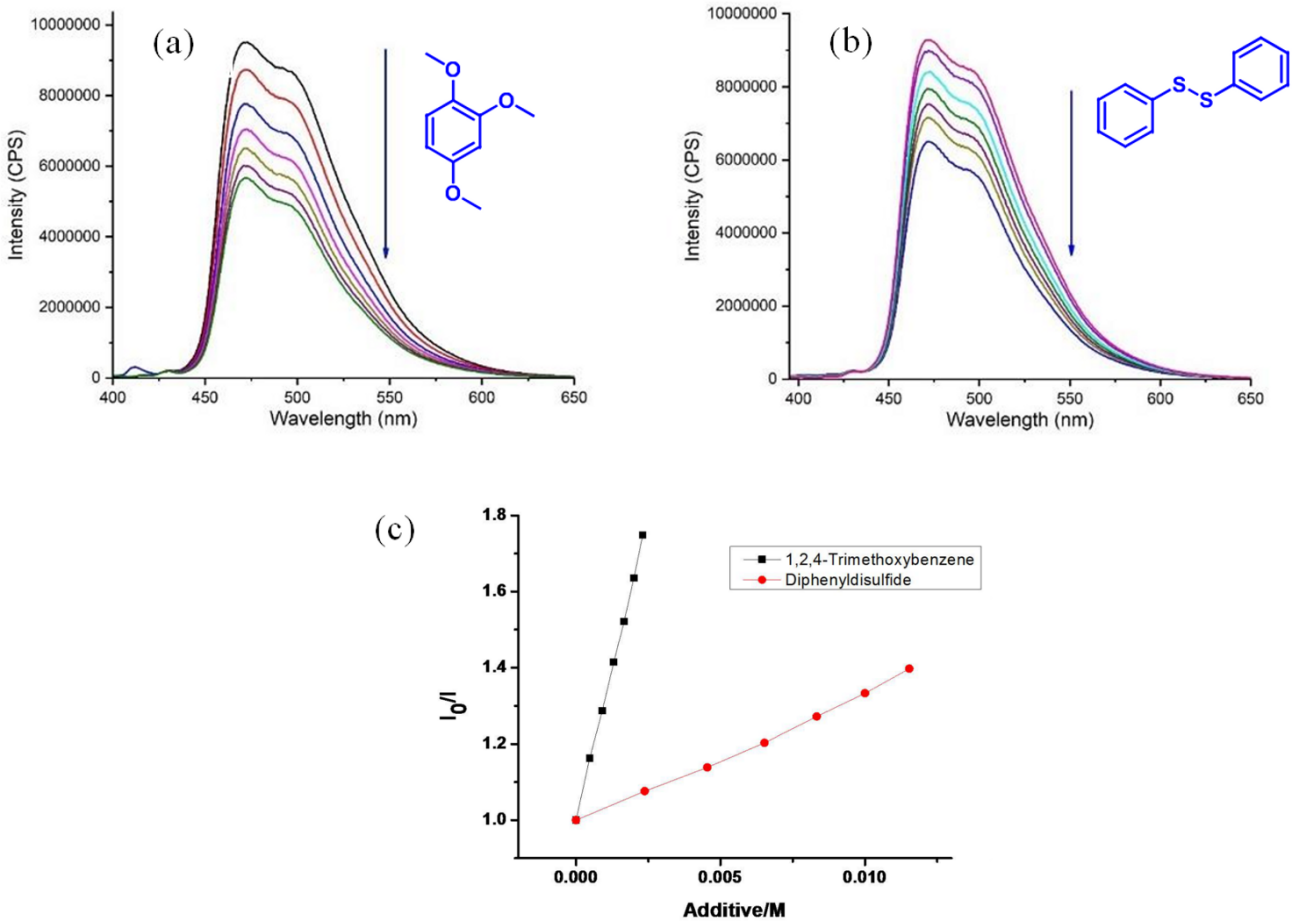
(a) Changes in the fluorescence spectra (in this case intensity, λ_Ex_ = 455 nm) of [Ir(dF(CF_3_)ppy)_2_(dtbpy)]PF_6_ upon the addition of 1,2,4-trimethoxybenzene in CH_3_CN. (b) Changes in the fluorescence spectra upon the addition of diphenyl disulfide in CH_3_CN. (c) Stern–Volmer quenching plot of iridium catalyst in the presence of 1,2,4-trimethoxybenzene and diphenyl disulfide. *K*_q_ (arene) = 318 ± 2.6 M^−1^ L and *K*_q_ (disulfide) = 36 ± 0.7 M^−1^ L.

Some spectroscopic investigations (Figure 3) gave valuable information about the mechanism of the photoredox catalytic cycle. The luminescence intensity of [Ir(dF(CF_3_)ppy)_2_(dtbpy)]PF_6_ was quenched upon successive addition of 1,2,4-trimethoxybenzene (oxidation potential 1.02 V vs SCE, Figure 3a). The values are similar to the estimated excited state oxidation potential of [Ir(dF(CF_3_)ppy)_2_(dtbpy)]PF_6_ ( +1.21 V vs SCE in acetonitrile). On the other hand, the luminescence was quenched negligible on addition of diphenyl disulfide (Figure 3b). Stern–Volmer quenching studies showed that the arene is quenched at a much higher rate than the disulfide (Figure 3c). This indicates that the oxidation of the arene is the key step in the C–H sulfenylation reaction. Anisole does not quench the luminescence of [Ir(dF(CF_3_)ppy)_2_(dtbpy)]PF_6_ and also did not give a sulfenylated product under our photocatalytic conditions. This is rationalized by the oxidation potential of anisole of 1.76 V vs SCE, which is higher than the estimated excited state oxidation potential of the photocatalyst.

**Scheme 5:**
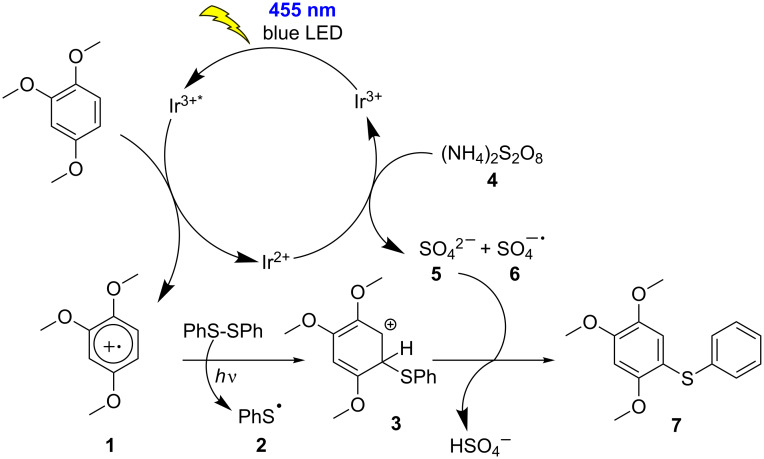
Proposed mechanism for visible light mediated direct C–H sulfenylation.

**Figure 4:**
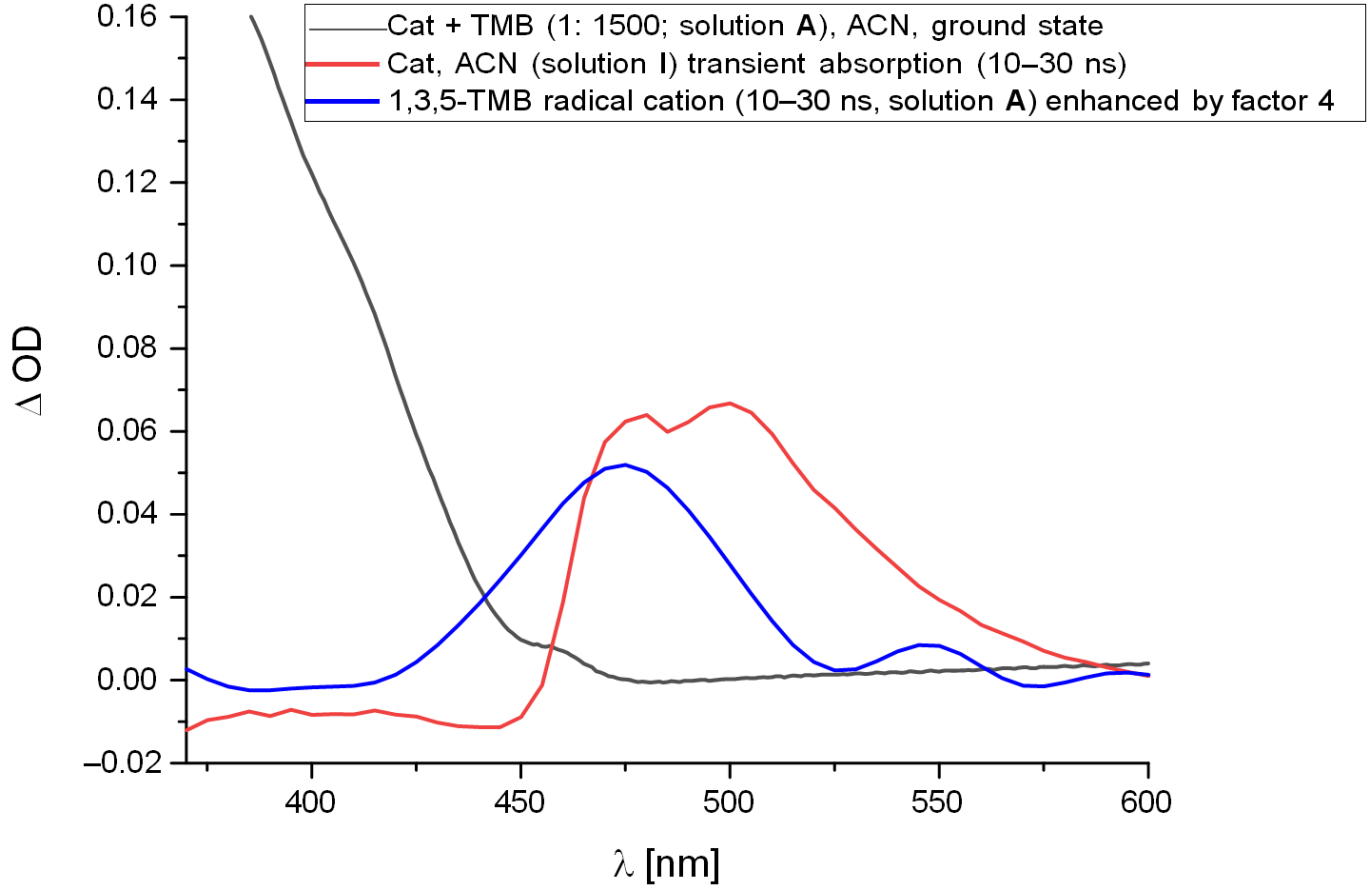
Black line: UV–vis spectrum of the degassed [Ir] + 1,3,5-TMB mixture (solution **A**) in ACN. Blue and red lines: Absorption spectra averaged over 10–30 ns after 355 nm laser-pulse with red being derived from the [Ir] solution **I** and representing the emission spectrum of the catalyst. Whereas, the blue line was obtained from the difference spectrum [Ir] and [Ir] + TMB and represents the transient absorption spectrum of 1,3,5-TMB^•+^ with λ_max_ of 473 nm (solution **A**).

To elucidate, if the 1,3,5-TMB radical cation (1,3,5-TMB^•+^) is formed indeed during the quenching process of the catalyst by 1,3,5-TMB (Scheme 5) ns-time-resolved transient absorption spectroscopy was used [50]. To allow for a decomposition of the multicomponent spectra we conducted laser flash photolysis (LFP) experiments on the single components (**I** = [Ir], **II** = 1,3,5-TMB, **III** = (PhS)_2_ in ACN, fpt-degassed) and the 2- and 3-component mixtures (**A** = [Ir]/[TMB] = 1:1500; **B** = [Ir]/[TMB]/[(PhS)_2_] = 1:1500:25 in ACN, fpt-degassed) [51]. Analyzing the single component solutions by LFP experiments with different time-resolutions and time-scales (400 ps/div, 10 ns/div, 10µs/div; *t*_max_ = 3.5 µs to 10 µs) provided information on photophysics and photochemistry of the single reactants. Figure 4 shows the strong luminescence of the catalyst (red line) that overlaps with the transient absorption of the postulated 1,3,5-TMB^•+^ in the 2-component mixture **A** [52]. The half-life time τ_1/2_ of the catalyst’s emission was determined to be 1.5 µs (mono-exponential fit at 520 nm) and corresponds well with published data on related compounds [53–55]. The 1,3,5-TMB on its own did not show any transients initiated by the 355 nm pulse. (PhS)_2_ produced under 355 nm irradiation, a broad transient absorption from 300 nm to 390 nm that did not decline over the measurement time (up to 10 µs, see Supporting Information File 1). In the UV–vis spectra recorded after the LFP experiment a significant change in absorption in the 300–370 nm region was observed, indicating that probably a fragmentation of the disulfide bond took place due to the laser irradiation. Since this effect occurred also under reduced laser power (70% of the original 58 mJ/pulse) we restricted the current analysis to the two-component solution (**A**). Since 1,3,5-TMB did not show any transient formation in solution **II**, the deconvolution of the spectra of **A** were achieved with the help of spectra derived of **I**. Taking the difference spectra on different time intervals revealed a transient absorption spectrum that corresponds to literature data of 1,3,5-TMB^•+^ (Figure 4). 1,3,5-TMB^•+^ emerges within the first 20 ns and has a life-time of around 4 µs. The presence of TMB led to a slower decay kinetics at wavelength where both fluorescence of that catalyst and the transient 1,3,5-TMB^•+^ occur, for instance at 447 nm *k*_decay,_**_I_**/*k*_decay,_**_A_** = 1.4.

Based on the above experimental results, spectroscopic investigations and literature reports, we propose a photocatalytic mechanism (Scheme 5). Upon photoexcitation, [Ir(dF(CF_3_)ppy)_2_(dtbpy)]PF_6_ accepts an electron from the arene and converts it into the corresponding radical cation **1**. Ammonium persulfate present in the reaction mixture could oxidize the reduced photocatalyst and complete the catalytic cycle forming the sulfate dianion **5** and sulfate radical anion **6**. The phenyl sulfide radical **2** formed upon homolytic cleavage of diphenyl disulfide adds to the radical cation of the arene to form the unstable cationic intermediate **3**. Aromatization by deprotonation leads to the desired product **7**.

## Conclusion

In conclusion, we have developed a photocatalytic method for the synthesis of aryl sulfides via a radical–radical cation cross coupling of electron rich arenes with aryl and alkyl disulfides. The reaction proceeds at room temperature and avoids the use of prefunctionalized arenes.

## Supporting Information

File 1 Experimental details, LFP data and NMR spectra of all compounds.

File 2 X-ray crystallographic data for **3a** (CCDC 1847083), **3d** (CCDC 1847084), **3e** (CCDC 1847085 and **3i** (CCDC 1847086). These data can be obtained free of charge from The Cambridge Crystallographic Data Centre.
