# Supplementary material for: Synthesis of aryl sulfides via radical–radical cross coupling of electron-rich arenes using visible light photoredox catalysis
Source: Beilstein J Org Chem. 2018 Sep 27;14:2520–8. doi: 10.3762/bjoc.14.228 (PMC6176842; doi:10.3762/bjoc.14.228)
Supplement: File 1 — Experimental details, LFP data and NMR spectra of all compounds. [file Beilstein_J_Org_Chem-14-2520-s001.pdf]

Supporting Information
for
Synthesis of aryl sulfides via radical–radical cross coupling of electron-rich arenes using visible light photoredox catalysis

Amrita Das, Mitasree Maity, Simon Malcherek, Burkhard König* and Julia Rehbein*

Address: Department of Chemistry and Pharmacy, Institute of Organic Chemistry, University of Regensburg, Universitätsstraße 31, 93053 Regensburg, Germany

Email: Burkhard König* - Burkhard.Koenig@chemie.uni-regensburg.de
Julia Rehbein* - Julia.Rehbein@chemie.uni-regensburg.de

*Corresponding author

Experimental details, LFP data and NMR spectra of all compounds

Table of contents

General information……………………………………………………………………S1

Optimization reactions and control experiments………………………………………...S3

HRMS analysis and TEMPO adduct……………………………………………………..S5

UV–vis spectra of the catalyst and 1,3,5 trimethoxybenzene…………………………S7

Laser flash photolysis experiments……………………………………………………..S8

General procedure for C–H sulfenylation………………………………………………..S13

Crystallographic data……………………………………………………………………S20

^{1}H and ^{13}C NMR spectra………………………………………………………………S22

References………………………………………………………………………………S35

General information

Solvents and reagents were obtained from commercial sources and used without further purification. Spectroscopic grade DMF and DMSO were dried with 3 Å molecular sieves according to a reported procedure.[1] $[Ir(dF(CF_3)ppy)_2(dtbbpy)]PF_6$ was synthesized according to the general procedure.[2] Proton NMR spectra were recorded on a Bruker Avance 300 MHz spectrometer and 600 MHz spectrometer in $CDCl_3$ solution with internal solvent signal peak at 7.26 ppm. Carbon NMR were recorded at 75 MHz spectrometer and 151 MHz spectrometer in $CDCl_3$ solution and referenced to the internal solvent signal at 77.26 ppm. Proton NMR data are reported as follows: chemical shift (ppm), multiplicity (s = singlet, d = doublet, t = triplet, q = quartet, quint = quintet, dd = doublet of doublets, ddd = doublet of doublet of doublets, td = triplet of doublets, qd = quartet of doublets, m = multiplet, br. s. = broad singlet), and coupling constants (Hz). High resolution mass spectra (HRMS) were obtained from the central analytic mass spectrometry facilities of the Faculty of Chemistry and Pharmacy, Regensburg University and are reported according to the IUPAC recommendations 2013. All reactions were monitored by thin-layer chromatography using Merck silica gel plates 60 F254; visualization was accomplished with short wave length UV light (254 nm). UV–Vis and fluorescence measurements were performed with Varian Cary 50 UV–vis spectrophotometer and FluoroMax-4 spectrofluorometer, respectively. For laser flash experiments, UV–vis spectra were recorded using an analytikjena SPECORD 5 PLUS spectrometer. Laser-flash photolysis (LFP) experiments were conducted with a Nd:YAG-laser (Continuum, Surelight SL I-10) using its third harmonics (355 nm, 58 mW and 40 mW output) and an "Applied Photophysics" spectrometer with monochromator and 5 stage photomultiplier. Digitalization was achieved with a Tektronix MDO 3034 Oscilloscope (50 Ω for ns time-scales and 1 kΩ for µs time-scales). The system was operated with the Applied Photophysics kinetic workbench program "Pro-Data LKS" (version 3.0.1). The probe and pulse light were arranged in an orthogonal setup. Transient spectra were recorded with 10 000 data points in the range from 300 nm to 620 nm, omitting the area of the pump wavelength (340–370 nm) and with a spectral resolution of 5 nm. All recordings of transient spectra were done performing four independent runs of the same sample that were then averaged. Kinetic traces were obtained by using an automated base line correction "internal dual" settings with four averages.

Spectra were analyzed and converted using the “Pro-Data Viewer” software. For further analysis of transient spectra and kinetics Origin 2017 was used. Experiments were conducted in acetonitrile (Acros, “extra dry”) in a sealed, nitrogen flushed quartz cuvette with glass neck (10 mm by 10 mm). The solvent was analyzed by UV–vis spectroscopy and under the described LFP conditions to ensure its optical purity. Electrochemical studies were carried out under argon atmosphere. The measurements were performed in dimethylformamide (DMF) containing 0.1 M tetra-*n*-butylammonium tetrafluoroborate using ferrocene/ferrocenium (Fc/Fc+) as an internal reference. A glassy carbon electrode (working electrode), platinum wire (counter electrode), and silver wire (quasi-reference electrode) were employed. Spectroelectrochemical studies were carried out in an optically transparent thin layer electrochemical cell (OTTLE). Suitable crystals were selected and mounted on a MITIGEN holder oil on a GV1000/SuperNova, TitanS2 diffractometer or Atlas diffractometer. Using **Olex2**[3], the structure was solved with the **ShelXT**[4] structure solution program, using the Intrinsic Phasing solution method. The model was refined with ShelXL using Least Squares minimisation. Standard flash chromatography was performed using silica gel of particle size 40−63 μm using petroleum ether (boiling range: 42–62 °C) and ethylacetate (boiling point: 77.1°C) as the eluent. Photoreduction and annulation reactions were performed with 455 nm LEDs (OSRAM Oslon SSL 80 royal-blue LEDs (λ = 455 nm (± 15 nm), 3.5 V, 700 mA).

Optimization reactions and control experiments

Table S1:

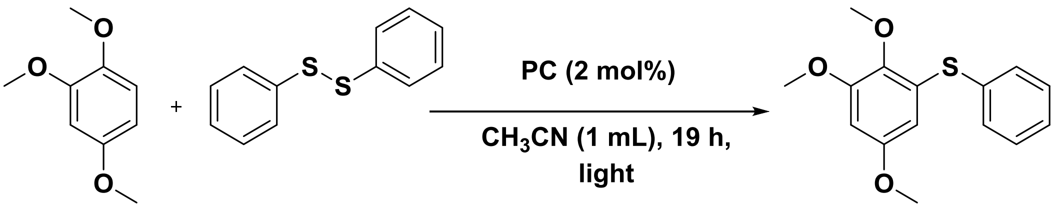

Entry	Photocatalyst	Oxidant	Light source	Air / N_2	Comments
1	Ir(dF-CF_3-ppy)$_2$(dtbpy)$^{3+}$	$(NH_4)_2S_2O_8$	455 nm	N_2	Full conversion to product was observed in 6 hours
2	Ir(dF-CF_3-ppy)$_2$(dtbpy)$^{3+}$	air	455 nm	air	Degradation of catalyst is observed upon irradiation
3	4-CzIPN	air	455 nm	air	Degradation of catalyst is observed upon irradiation
4	4-CzIPN	$(NH_4)_2S_2O_8$	455 nm	N_2	30% conversion of the starting material was observed
5	Eosin Y	air	530 nm	air	PC bleached in 2 hours
6	Eosin Y	$(NH_4)_2S_2O_8$	530 nm	N_2	Degradation of catalyst is observed upon irradiation
7	Eosin Y-Na_2	air	530 nm	air	Degradation of catalyst is observed upon irradiation
8	Eosin Y-Na_2	$(NH_4)_2S_2O_8$	530 nm	N_2	10% conversion of the starting material was observed

Table S2:

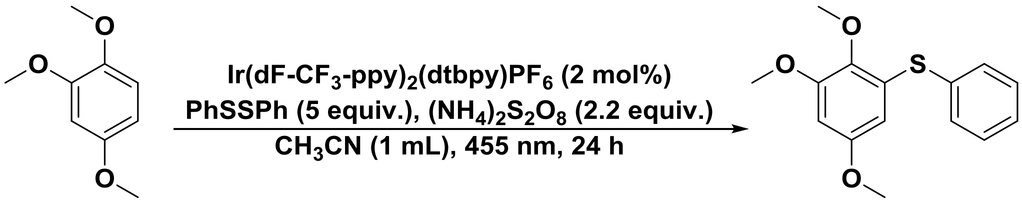

Entry	Photocatalyst	Oxidant	N_2/ air	Light/ dark	Comments
1	✓	✓	N_2	dark	Only Starting material, no conversion
2	✓	✓	N_2	455 nm	Full conversion to product
3	✓	✓	air	dark	Only Starting material, some oxidative side reactions of the disulphide, no product formation
4	✓	✓	air	455 nm	Catalyst bleached immediately
5	✓	✗	N_2	dark	Only Starting material, no conversion
6	✓	✗	N_2	455 nm	10% conversion by GC
7	✓	✗	air	dark	Only Starting material, no conversion
8	✓	✗	air	455 nm	Catalyst bleached immediately

HRMS analysis of the TEMPO adduct

A solution of 1,2,4 trimethoxybenzene in CH_3CN with 2 mol % $(Ir[dF(CF_3)ppy]_2(dtbpy))PF_6$, 2.2 equivalents $(NH_4)_2S_2O_8$ and 2 equivalents TEMPO was irradiated with a 455 nm LED under N_2 at 25 ºC. The reaction mixture was analyzed by HRMS, which showed the exact molecular ion indicating the formation of the proposed TEMPO adduct to the aryl radical cation intermediate.

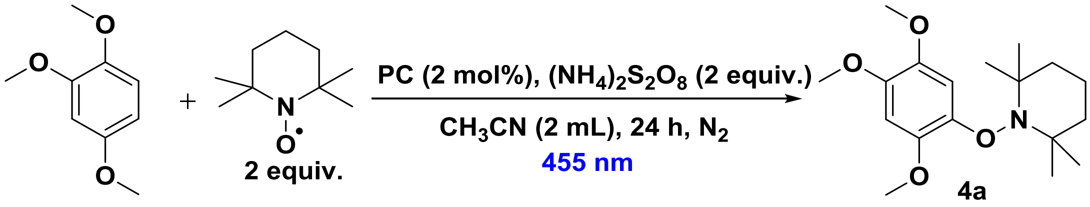

HRMS [M+H]+ $C_{18}H_{29}NO_4$ calculated 324.2175 found 324.2169

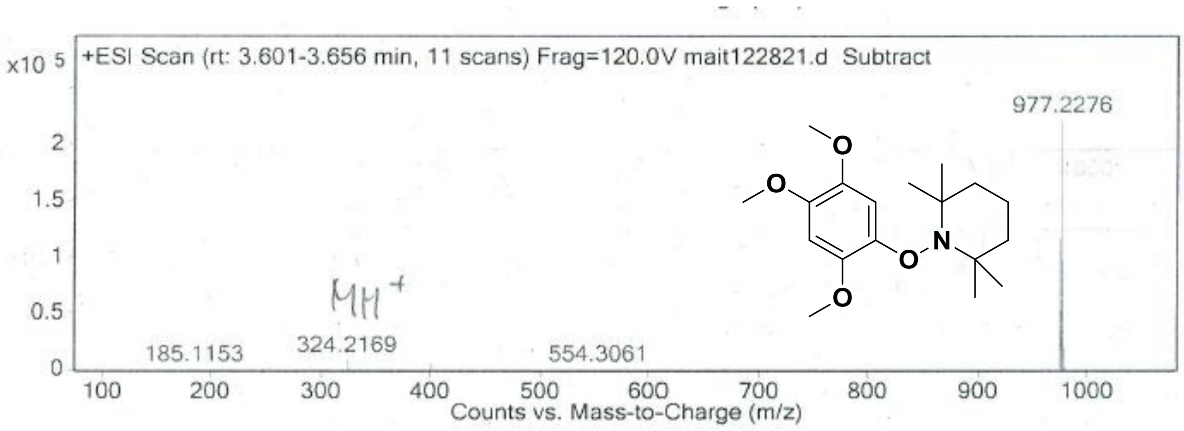

A solution of diphenyl disulfide in CH_3CN with 2 mol % $(Ir[dF(CF_3)ppy]_2(dtbpy))PF_6$ and 2 equivalents TEMPO was irradiated with a 455 nm LED under N_2 at 25 ºC. The reaction mixture was analyzed by HRMS, which showed the exact molecular ion indicating the formation of the proposed TEMPO adduct to the aryl radical cation intermediate.

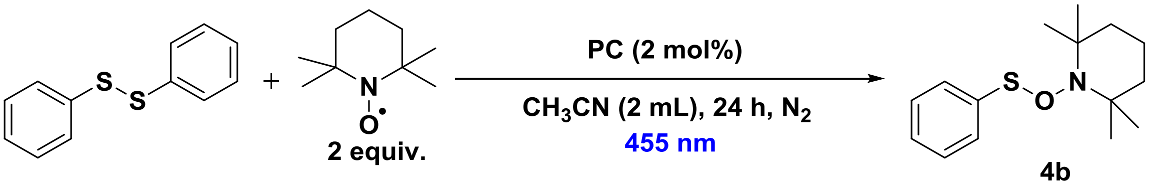

HRMS [M+H]+ $C_{15}H_{23}NOS$ calculated 266.1579 found 266.1577

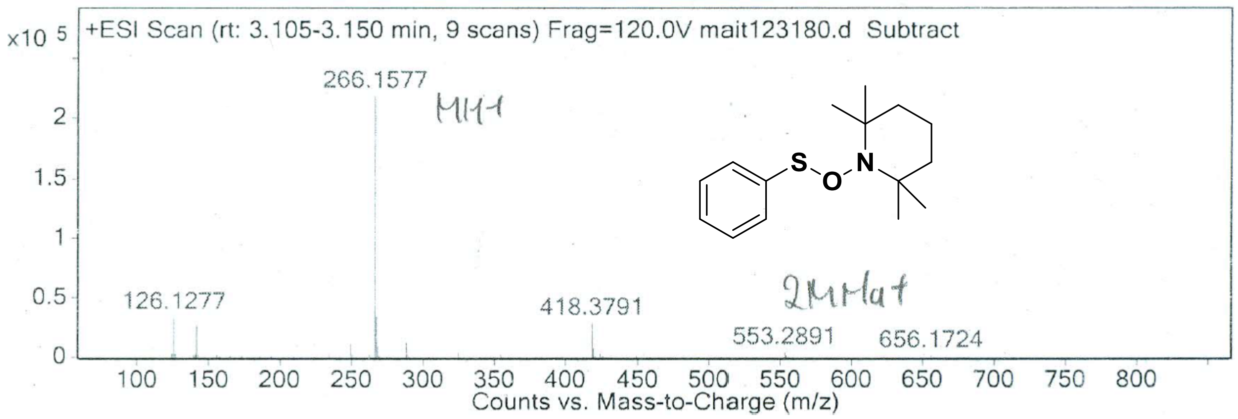

A solution of diphenyl disulfide in CH_3CN and 2 equivalents TEMPO was irradiated with a 455 nm LED under N_2 at 25 ºC. The reaction mixture was analyzed by HRMS, which showed the exact molecular ion indicating the formation of the proposed TEMPO adduct to the aryl radical cation intermediate.

HRMS [M+H]+ $C_{15}H_{23}NOS$ calculated 266.1579 found 266.1577

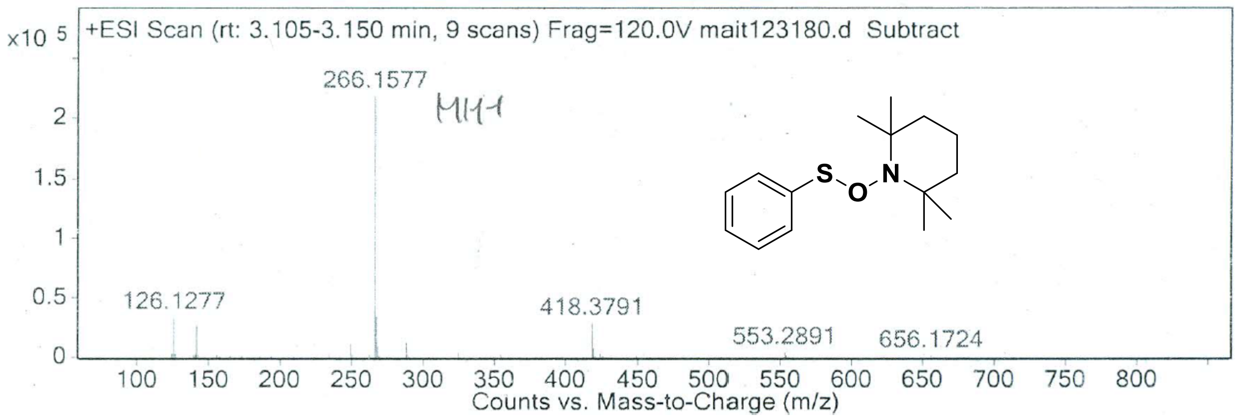

UV–vis spectra of the catalyst and 1,3,5 trimethoxybenzene

Prior to experiments samples have been degassed by threefold execution of the freeze-pump-thaw procedure. Concentration of reagents in samples was: (Ir[dF(CF_3)ppy]$_2$(dtbpy))PF_6 = $3.4 \cdot 10^{-5}$ mol/L (**solution I**) TMB = $50.5 \cdot 10^{-3}$ mol/L (**solution II**) and diphenyl disulfide = $8.1 \cdot 10^{-4}$ mol/L (**solution III**). This is equal to equivalents of 1, 1500 and 25 respectively in the reaction mixtures **A** (cat + TMB) and **B** [cat + TMB + $(PhS)_2$]. During and after measurements, ground states of the samples were confirmed *via* UV-Vis spectroscopy to ensure no change in the sample had taken place. The UV-Vis spectrum of the (Ir[dF(CF_3)ppy]$_2$(dtbpy))PF_6-catalyst (cat, **Figure 1**) has a significant absorbance at 355 nm mandatory for the LFP experiments. 1,3,5-Trimehoxybenzene (TMB) is transparent at the pump wavelength and hence could be used in high excess (1500 equiv). The absorption spectrum of the mixture of catalyst and TMB was equal to the catalyst spectrum. Although diphenyl disulfide does not absorb significantly at the pump wavelength (355 nm) a change in the UV-vis spectrum occurred due to the LFP experiments even without catalyst and substrate.

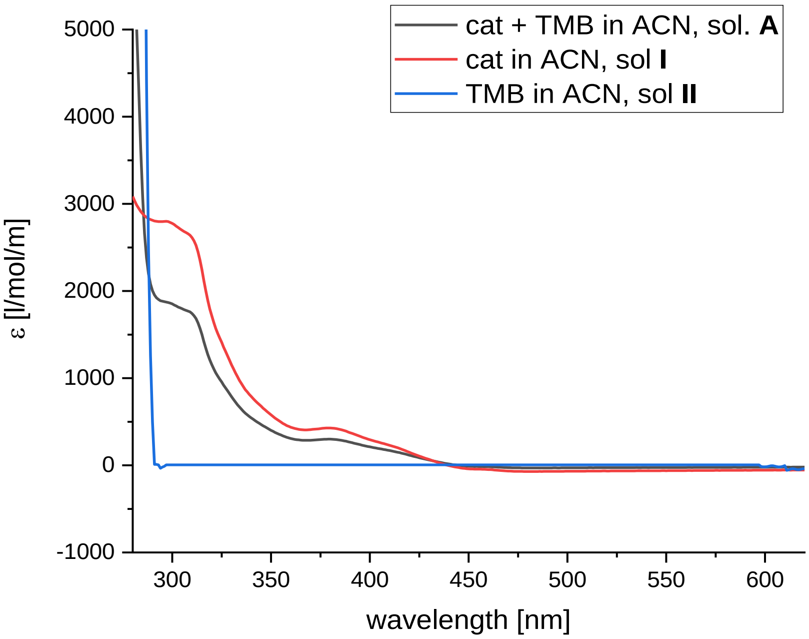

Figure S1: UV–vis of single components: catalyst, 1,3,5-TMB and mixture **A** (cat + 1500 equiv 1,3,5-TMB) in degassed acetonitrile.

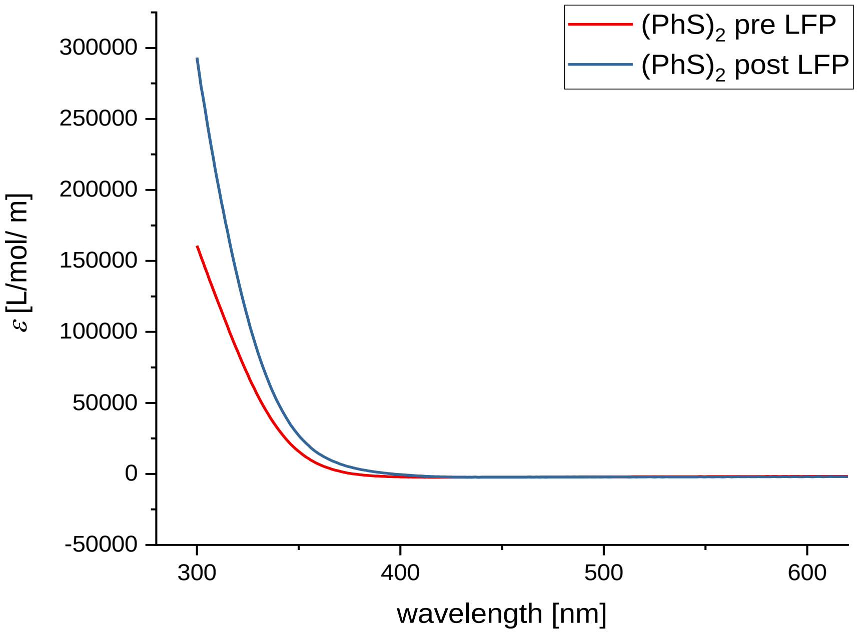

Figure S2: UV–vis spectra of diphenyl disulfide in degassed acetonitrile before (red line) and after (blue line) single laser flash experiment.

Laser flash photolysis experiments

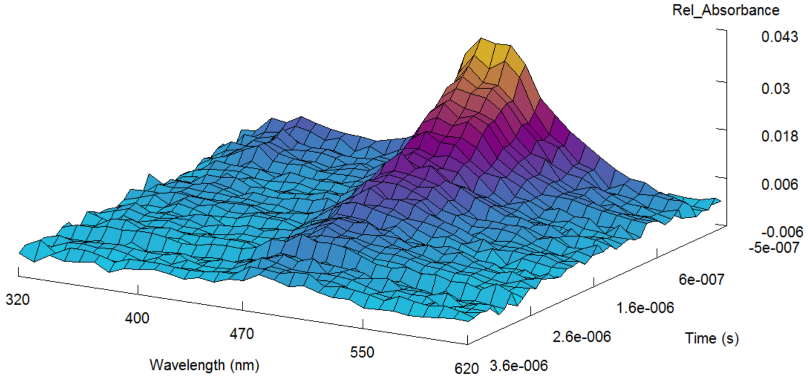

Figure S3: Spectra kinetics (400 ps/div), catalyst solution **I**.

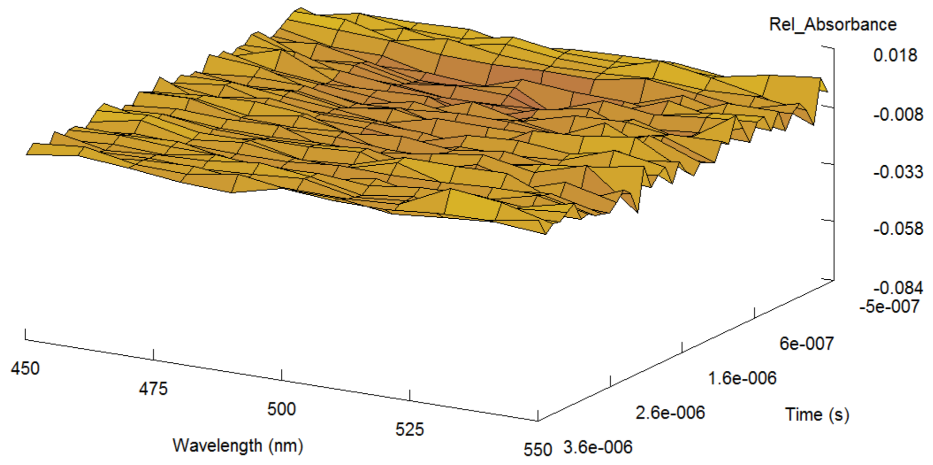

Figure S4: Spectra kinetics (400 ps/div), 1,3,5-TMB solution **II**.

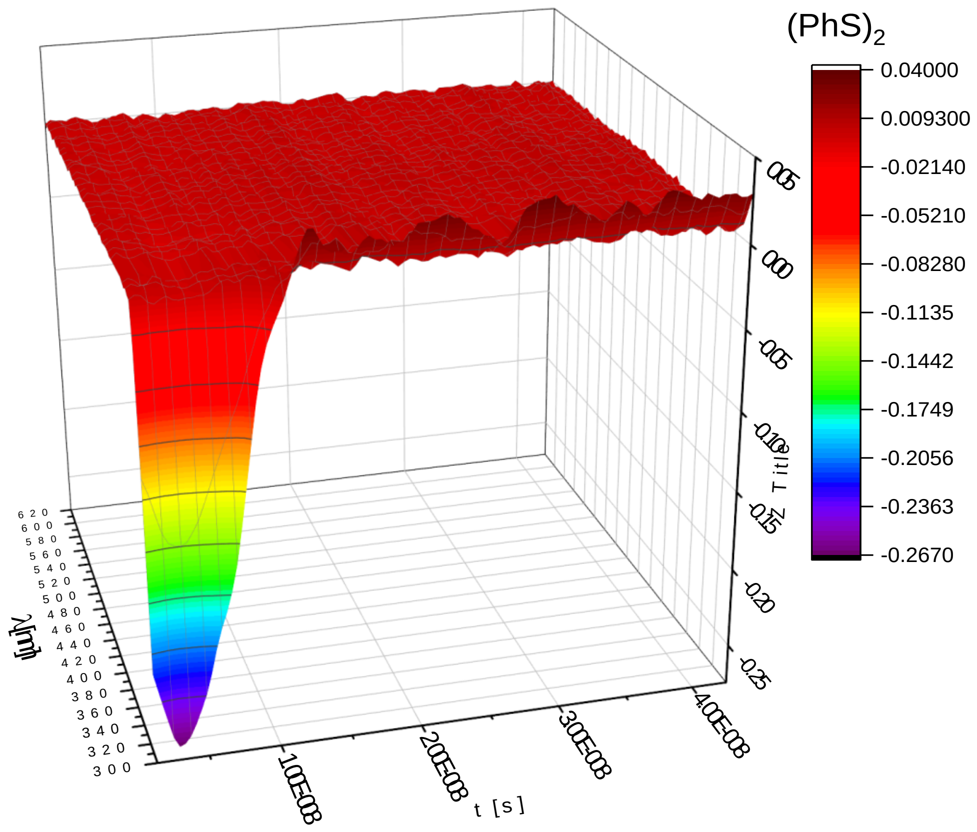

Figure S5: Spectra kinetics (400 ps/div), $(PhS)_2$ solution **III**.

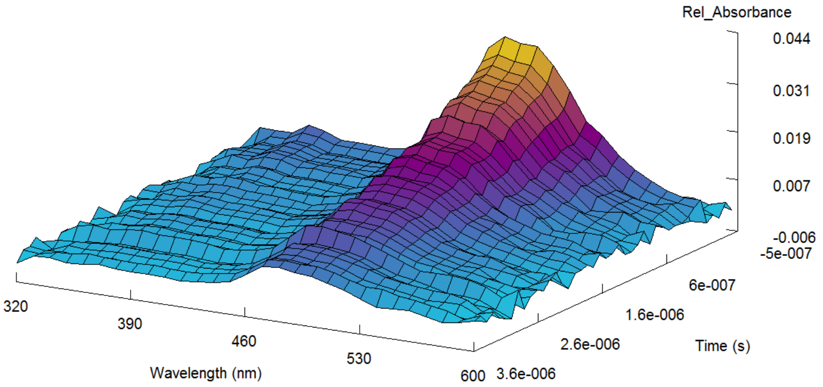

Figure S6: Spectra kinetics (400 ps/div), solution **A**.

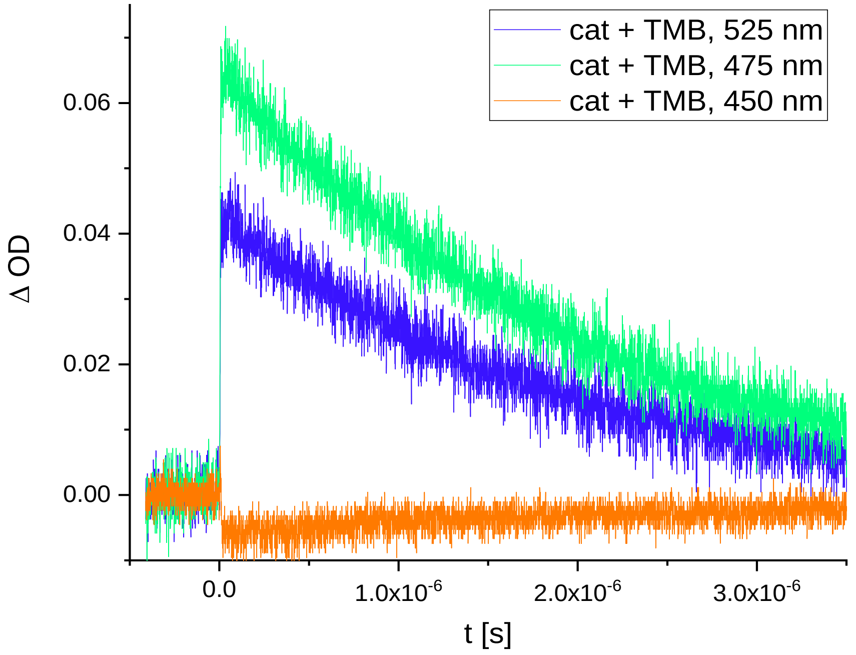

Figure S7: Kinetic traces recorded of (Ir[dF(CF_3)ppy]$_2$(dtbpy))PF_6 - TMB mixture at 450 nm, 475 nm and 525 nm in degassed ACN (solution **A**) with 400 ps /div.

Figure S6 and S**7** show the differences at the indicative wavelength for transient species. At 475 nm the maximum of the TMB•+ is observed leading to a higher change in optical density in solution **A** compared to solution **I**. The half-life of TMB•+ was determined of the decay traces in **Figure S8** to be 2.2 μs which is comparable to what has been observed for related compounds.[3] At 525 nm only the catalyst's emission should contribute significantly to the change in optical density (Δ OD) and indeed no proper kinetic signature is observed in the kinetic trace derived of the differences spectrum at 525 nm (**Figure 9**).

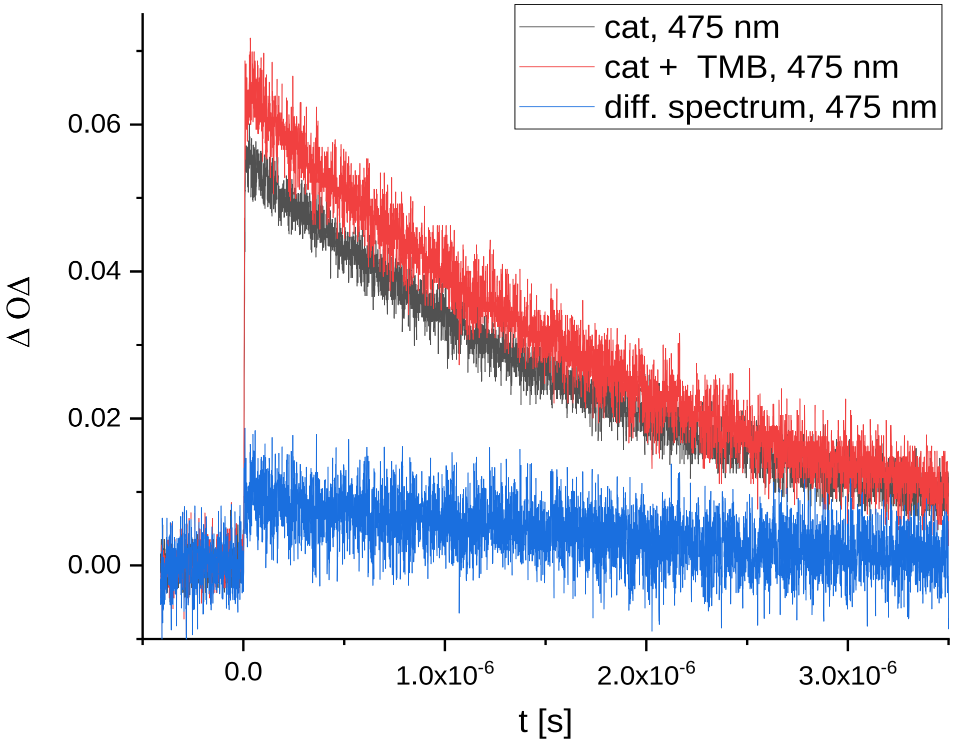

Figure S8: Kinetic trace at 475 nm recorded of solution **I** and **A** in degassed ACN (A) with 400 ps /div and the trace derived of subtracting the traces. Monoexponential fits of these decays were obtained: $k_I = 4.86 \pm 0.04\ s^{-1}$, $k_A = 4.61 \pm 0.04\ s^{-1}$, $k_{Diff} = 3.2 \pm 0.2\ s^{-1}$. With the latter describing the decay of TMB•+.

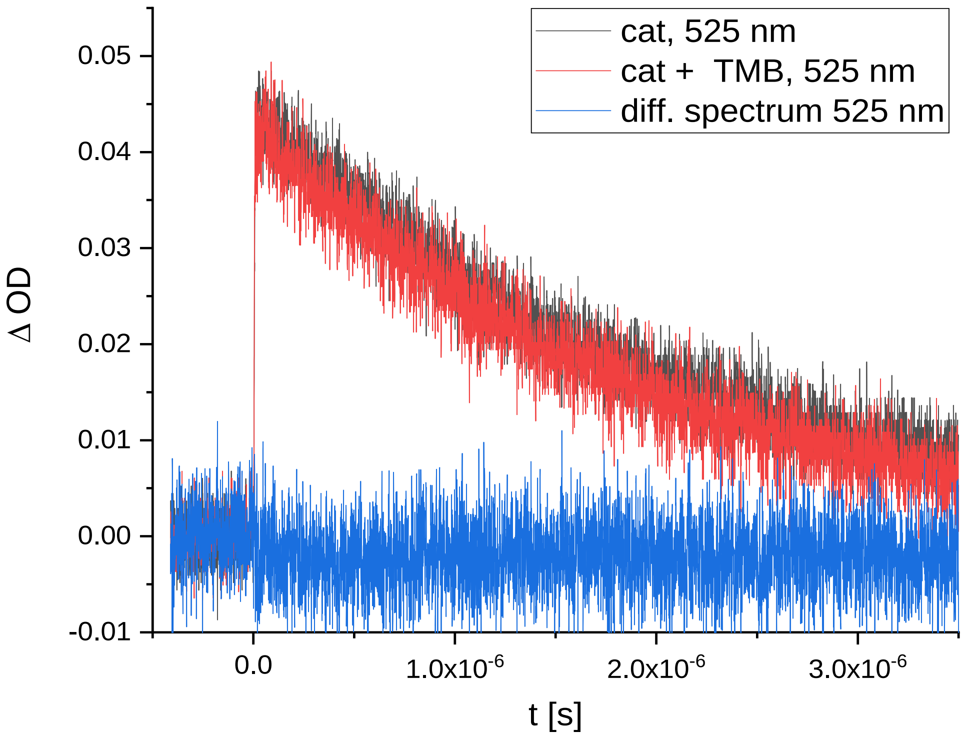

Figure S9: Mono-exponential fit of the kinetic trace at 525 nm recorded of $Ir[dF(CF_3)ppy]_2(dtbpy))PF_6$ + TMB in degassed ACN (**A**) with 400 ps /div.

General procedure for C–H sulfenylation

In a 5 mL snap vial with magnetic stirring bar, the arene (0.1 mmol, 1.0 equiv), the aryl disulfide (2.0 equiv), $(NH_4)_2S_2O_8$ (1.7 equiv) and $(Ir[dF(CF_3)ppy]_2(dtbpy))PF_6$ (0.002 mmol, 0.02 equiv) were added. Dry CH_3CN (2.0 mL) were added under N_2 and the mixture was degassed by "pump-freeze-thaw" cycles (×3) via a syringe needle. This reaction mixture was irradiated through the plane bottom side of the snap vial using a 455 nm LED under nitrogen at 25 °C. The reaction progress was monitored by GC analysis. After 6 h to 24 h of irradiation, the reaction mixture was transferred to separating funnel, diluted with ethyl acetate and washed with 15 mL of water. The aqueous layer was washed three times (3 × 15 mL) with ethyl acetate. The combined organic phases were dried over $MgSO_4$, filtered and concentrated in vacuum. Purification of the crude product was achieved by flash column chromatography using petrol ether/ethyl acetate as eluent.

Synthesis of (2,4,6-trimethoxy-1,3-phenylene)bis(phenylsulfane) (3a)[5]

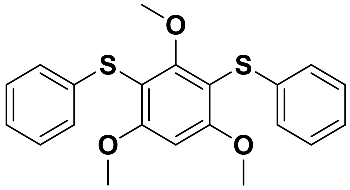

The compound was prepared according to the general procedure using 16.8 mg of 1,3,5-trimethoxybenzene, 2.2 mg of $[Ir(dF(CF_3)ppy)_2(dtbpy)]PF_6$, 43.6 mg of diphenyl disulfide, 40 mg of $(NH_4)_2S_2O_8$ and 2.0 mL dry CH_3CN. The reaction mixture was irradiated for 6 hours under N_2. Purification of the crude product was achieved by flash column chromatography using petrol ether/ethylacetate (85:15) as eluent to yield the desired compound as white solid (20.4 mg, 53%).

^{1}H NMR ($CDCl_3$, 300 MHz, ppm): δ 7.25-7.15 (m, 4H), 7.08-7.05 (m, 4H), 6.43 (s, 1H), 3.87 (s, 6H), 3.76 (s, 3H).

^{13}C NMR ($CDCl_3$, 75 MHz, ppm): δ 166.3, 163.7, 138.4, 128.8, 126.2, 125.0, 106.8, 92.5, 62.5, 56.5.

HRMS [$M^{+\cdot}$] $C_{21}H_{20}O_3S_2$ calculated 384.0848 found 384.0852.

Synthesis of (2,4,6-trimethoxy-1,3-phenylene)bis(p-tolylsulfane) (3b)

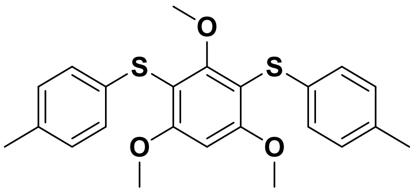

The compound was prepared according to the general procedure using 16.8 mg of 1,3,5-trimethoxybenzene, 2.2 mg of [Ir(dF(CF$_3$)ppy)$_2$(dtbpy)]PF$_6$, 49.2 mg of 1,2-di-*p*-tolyldisulfane, 40 mg of $(NH_4)_2S_2O_8$ and 2.0 mL dry CH_3CN. The reaction mixture was irradiated for 6 h under N_2. Purification of the crude product was achieved by flash column chromatography using petrol ether/ ethylacetate (85:15) as eluent to yield the desired compound as white solid (23.1 mg, 56%).

^{1}H NMR ($CDCl_3$, 300 MHz, ppm): δ 6.99 (s, 8H), 6.41 (s, 1H), 3.86 (s, 6H), 3.76 (s, 3H), 2.26 (s, 6H).

^{13}C NMR ($CDCl_3$, 75 MHz, ppm): δ 166.1, 163.5, 134.8, 129.6, 126.6, 107.4, 92.5, 62.5, 56.5, 21.1.

HRMS [M$^{+\cdot}$] $C_{23}H_{24}O_3S_2$ calculated 412.1161 found 412.1170.

Synthesis of (2,4,6-trimethoxy-1,3-phenylene)bis((4-methoxyphenyl)sulfane) (3c)

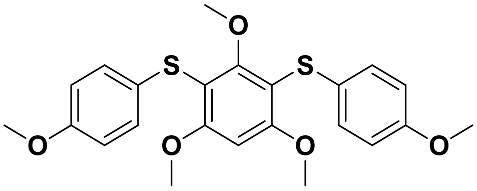

The compound was prepared according to the general procedure using 16.8 mg of 1,3,5-trimethoxybenzene, 2.2 mg of [Ir(dF(CF$_3$)ppy)$_2$(dtbpy)]PF$_6$, 55.6 mg of 1,2-bis(4-methoxyphenyl)disulfane, 40 mg of $(NH_4)_2S_2O_8$ and 2.0 mL dry CH_3CN. The reaction mixture was irradiated for 6 hours under N_2. Purification of the crude product was achieved by flash column chromatography using petrol ether/ ethylacetate (85:15) as eluent to yield the desired compound as white solid (24.0 mg, 54%).

^{1}H NMR ($CDCl_3$, 300 MHz, ppm): δ 7.11-7.08 (m, 4H), 6.75-6.72 (m, 4H), 6.36 (s, 1H), 3.84 (s, 6H), 3.79 (s, 3H), 3.73 (s, 6H).

^{13}**C NMR** ($CDCl_3$, 75 MHz, ppm): δ 165.6, 163.1, 158.0, 129.3, 128.9, 114.5, 108.6, 92.6, 62.5, 56.5, 55.5.

HRMS [M+H]+ $C_{23}H_{24}O_5S_2$ calculated 445.1138 found 445.1140.

Synthesis of 2,4,6-trimethoxyphenyl dimethylcarbamodithioate (3d)

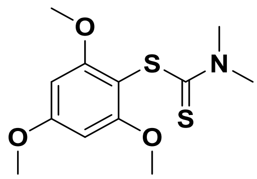

The compound was prepared according to the general procedure using 16.8 mg of 1,3,5-trimethoxybenzene, 2.2 mg of [Ir(dF(CF_3)ppy)$_2$(dtbpy)]PF_6, 47.8 mg of tetramethylthiuramdisulfid, 40 mg of $(NH_4)_2S_2O_8$ and 2.0 mL dry CH_3CN. The reaction mixture was irradiated for 24 hours under N_2. Purification of the crude product was achieved by flash column chromatography using petrol ether/ ethylacetate (80:20) as eluent to yield the desired compound as white solid (19.6 mg, 68%).

^{1}**H NMR** ($CDCl_3$, 300 MHz, ppm): δ 6.21 (s, 2H), 3.85 (s, 3H), 3.83 (s, 6H), 3.54 (s, 6H).

^{13}**C NMR** ($CDCl_3$, 75 MHz, ppm): δ 197.6, 164.0, 162.7, 100.1, 91.4, 56.6, 55.5.

HRMS [M+H]+ $C_{12}H_{17}NO_3S_2$ calculated 288.0723 found 288.0727.

Synthesis of (4,6-dimethoxy-1,3-phenylene)bis(phenylsulfane) (3e)

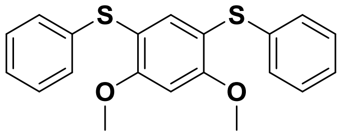

The compound was prepared according to the general procedure using 12.7 µL of 1,2-dimethoxybenzene, 2.2 mg of (Ir[dF(CF_3)ppy]$_2$(dtbpy))PF_6, 43.6 mg of diphenyl disulfide, 40 mg of $(NH_4)_2S_2O_8$ and 2.0 mL dry CH_3CN. The reaction mixture was irradiated for 6 hours under N_2. Purification of the crude product was achieved by flash column chromatography using petrol ether/ ethylacetate (85:15) as eluent to yield the desired compound as yellowish white solid (14.2 mg, 40%).

^{1}**H NMR** ($CDCl_3$, 300 MHz, ppm): δ 7.39 (s, 1H), 7.25-7.10 (m, 10H), 6.55 (s, 1H), 3.88 (s, 6H).

^{13}**C NMR** ($CDCl_3$, 75 MHz, ppm): δ 160.82, 141.35, 136.74, 129.01, 128.59, 126.04, 113.44, 96.22, 56.39.

HRMS[$M^{+\cdot}$] $C_{20}H_{18}O_2S_2$ calculated 354.0742 found 354.0743.

Synthesis of phenyl(2,4,5-trimethoxyphenyl)sulfane (3f)[6]

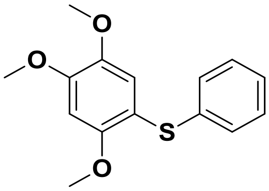

The compound was prepared according to the general procedure using 15.0 μL of 1,2,4-trimethoxybenzene, 2.2 mg of $[Ir(dF(CF_3)ppy)_2(dtbpy)]PF_6$, 43.6 mg of diphenyl disulfide, 40 mg of $(NH_4)_2S_2O_8$ and 2.0 mL dry CH_3CN. The reaction mixture was irradiated for 6 hours under N_2. Purification of the crude product was achieved by flash column chromatography using petrol ether/ ethylacetate (85:15) as eluent to yield the desired compound as pale yellow liquid (11.2 mg, 40%).

^{1}H NMR ($CDCl_3$, 300 MHz, ppm): δ 7.26-7.22 (m, 3H), 7.20-7.11 (m, 2H), 6.95 (s, 1H), 6.59 (s, 1H), 3.93 (s, 3H), 3.82 (s, 3H), 3.78 (s, 3H)

^{13}C NMR ($CDCl_3$, 75 MHz, ppm): δ 154.6, 150.9, 143.6, 137.9, 129.0, 127.7, 119.0, 110.0, 98.1, 57.2, 56.7, 56.3.

HRMS[$M^{+\cdot}$] $C_{15}H_{16}O_3S$ calculated 276.0814 found 276.0817.

Synthesis of p-tolyl(2,4,5-trimethoxyphenyl)sulfane (3g)[6]

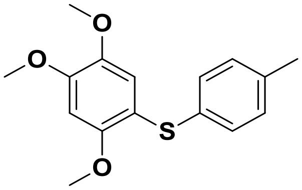

The compound was prepared according to the general procedure using 15.0 μL of 1,2,4-trimethoxybenzene, 2.2 mg of $[Ir(dF(CF_3)ppy)_2(dtbpy)]PF_6$, 49.2 mg of 1,2-di-*p*-tolyldisulfane, 40 mg of $(NH_4)_2S_2O_8$ and 2.0 mL dry CH_3CN. The reaction mixture was irradiated for 24 hours under N_2. Purification of the crude product was achieved by flash column chromatography using petrol ether/ ethylacetate (85:15) as eluent to yield the desired compound as white solid (12.5 mg, 43%).

^{1}H NMR ($CDCl_3$, 300 MHz, ppm): δ 7.10-7.02 (m, 4H), 6.87 (s, 1H), 6.57 (s,1H), 3.91 (s, 3H), 3.81 (s, 3H), 3.75 (s, 3H), 2.28 (s, 3H).

^{13}C NMR ($CDCl_3$, 75 MHz, ppm): δ 154.0, 150.0, 143.5, 135.9, 133.6, 129.8, 128.8, 118.2, 98.1, 57.1, 56.7, 56.3, 21.1.

HRMS[$M^{+\cdot}$] $C_{16}H_{18}O_3S$ calculated 290.0971 found 290.0977.

Synthesis of (4-methoxyphenyl)(2,4,5-trimethoxyphenyl)sulfane (3h)[7]

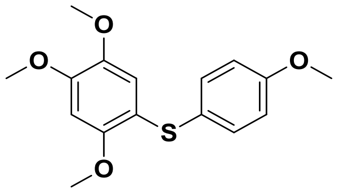

The compound was prepared according to the general procedure using 15.0 µL of 1,2,4-trimethoxybenzene, 2.2 mg of [Ir(dF(CF_3)ppy)$_2$(dtbpy)]PF_6, 55.6 mg of 1,2-bis(4-methoxyphenyl)disulfane, 40 mg of $(NH_4)_2S_2O_8$ and 2.0 mL dry CH_3CN. The reaction mixture was irradiated for 6 hours under N_2. Purification of the crude product was achieved by flash column chromatography using petrol ether/ ethylacetate (86:14) as eluent to yield the desired compound as pale yellow liquid (26.0 mg, 85%).

^{1}H NMR ($CDCl_3$, 300 MHz, ppm): δ 7.25-7.22 (m, 2H), 6.83-6.80 (m, 2H), 6.71 (s, 1H), 6.53 (s, 1H), 3.87 (s, 3H), 3.82 (s, 3H), 3.76 (s, 3H), 3.69(s, 3H).

^{13}C NMR ($CDCl_3$, 75 MHz, ppm): δ 159.0, 152.8, 149.7, 143.6, 132.5, 126.4, 116.5, 114.8, 114.4, 98.1, 57.0, 56.7, 56.3, 55.5.

HRMS[$M^{+\cdot}$] $C_{16}H_{18}O_4S$ calculated 306.0920 found 306.0926.

Synthesis of 2,4,5-trimethoxyphenyl dimethylcarbamodithioate (3i)

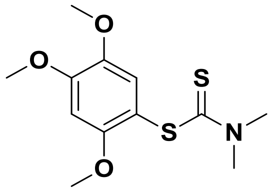

The compound was prepared according to the general procedure using 15.0 µL of 1,2,4-trimethoxybenzene, 2.2 mg of [Ir(dF(CF_3)ppy)$_2$(dtbpy)]PF_6, 47.8 mg of tetramethylthiuramdisulfid, 40 mg of $(NH_4)_2S_2O_8$ and 2.0 mL dry CH_3CN. The reaction mixture was irradiated for 24 hours under N_2. Purification of the crude product was achieved by flash

column chromatography using petrol ether/ ethylacetate (80:20) as eluent to yield the desired compound as yellow solid (12.7 mg, 44%).

^{1}H NMR ($CDCl_3$, 300 MHz, ppm): δ 6.89 (s, 1H), 6.61 (s, 1H), 3.94 (s, 3H), 3.86 (s, 3H), 3.84 (s, 3H), 3.54 (d, *J* = 11.1 Hz, 6H).

^{13}C NMR ($CDCl_3$, 75 MHz, ppm): δ 197.8, 156.0, 152.4, 143.3, 121.1, 109.5, 97.7, 57.3, 56.6, 56.1, 45.9, 42.1.

HRMS [M+H]+ $C_{12}H_{17}NO_3S_2$ calculated 288.0723 found 288.0724.

Synthesis of *N*-(4-ethoxy-3-(phenylthio)phenyl)acetamide (3j)

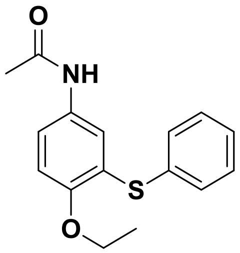

The compound was prepared according to the general procedure using 17.9 mg of *N*-(4-ethoxyphenyl)acetamide, 2.2 mg of [Ir(dF(CF_3)ppy)$_2$(dtbpy)]PF_6, 43.6 mg of diphenyl disulfide, 40 mg of $(NH_4)_2S_2O_8$ and 2.0 mL dry CH_3CN. The reaction mixture was irradiated for 24 hours under N_2. Purification of the crude product was achieved by flash column chromatography using petrol ether/ ethylacetate (85:15) as eluent to yield the desired compound as white solid (5.8 mg, 20%)

^{1}H NMR ($CDCl_3$, 600 MHz, ppm): δ 8.23 (d, *J* = 9 Hz, 1H), 7.86 (bs, 1H), 7.33-7.29 (m, 1H), 7.25-7.24 (m, 1H), 7.17 (t, *J* = 7.2 Hz, 1H), 7.11 (d, *J* = 7.5 Hz, 2H), 7.07 (d, *J* = 2.8 Hz, 1H), 6.96 (dd, *J* = 9 Hz, 2.8 Hz, 1H), 3.98 (q, *J* = 6.9 Hz, 2H), 2.00 (s, 3H), 1.38 (t, *J* = 7 Hz, 3H).

^{13}C NMR ($CDCl_3$, 150 MHz, ppm): δ 168.2, 155.5, 135.6, 133.0, 129.5, 127.7, 126.6, 122.8, 122.1, 121.4, 116.8, 64.0., 24.6, 14.9.

HRMS [M+H]+ $C_{16}H_{17}NO_2S$ calculated 288.1053 found 288.1046.

Synthesis of 1-phenyl-3-(phenylthio)pyrrolidine-2,5-dione (3k)

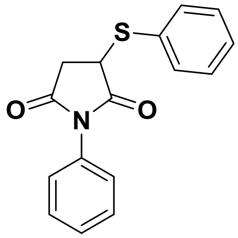

The compound was prepared according to the general procedure using 17.3 mg of 1-phenyl-3-(phenylthio)pyrrolidine-2,5-dione, 2.2 mg of [Ir(dF(CF_3)ppy)$_2$(dtbpy)]PF_6, 43.6 mg of diphenyl disulfide, 40 mg of $(NH_4)_2S_2O_8$ and 2.0 mL dry CH_3CN. The reaction mixture was irradiated for 24 hours under N_2. Purification of the crude product was achieved by flash column chromatography using petrol ether/ ethylacetate (85:15) as eluent to yield the desired compound as white solid (16.3 mg, 58%).

^{1}H NMR ($CDCl_3$, 600 MHz, ppm): δ 7.59-7.56 (m, 2H), 7.45-7.36 (m, 7H), 7.06-7.03 (m, 2H).

^{13}C NMR ($CDCl_3$, 151 MHz, ppm): δ 174.7, 173.7, 135.3, 133.8, 131.7, 129.9, 129.7, 129.3, 129.0, 126.5, 44.3, 36.6.

HRMS [M+H]+ $C_{16}H_{13}NO_2S$ calculated 284.0740 found 284.0734.

Synthesis of (2-methoxynaphthalen-1-yl)(phenyl)sulfane (3l)

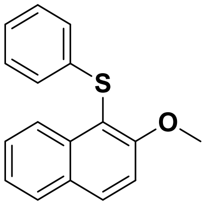

The compound was prepared according to the general procedure using 15.8 mg of (2-methoxynaphthalen-1-yl)(phenyl)sulfane, 2.2 mg of [Ir(dF(CF_3)ppy)$_2$(dtbpy)]PF_6, 43.6 mg of diphenyl disulfide, 40 mg of $(NH_4)_2S_2O_8$ and 2.0 mL dry CH_3CN. The reaction mixture was irradiated for 24 hours under N_2. Purification of the crude product was achieved by flash column chromatography using petrol ether/ ethylacetate (85:15) as eluent to yield the desired compound as white solid (8 mg, 30%).

^{1}H NMR ($CDCl_3$, 300 MHz, ppm): δ 8.48 (d, J = 8.4 Hz, 1H), 7.98 (d, J = 9.0 Hz, 1H), 7.84 (d, J = 8.1 Hz, 1H), 7.54-7.48 (m, 1H), 7.42-7.37 (m, 2H), 7.17-7.14 (m, 5H), 3.97 (s, 3H).

^{13}C NMR ($CDCl_3$, 75 MHz, ppm): δ 159.4, 138.3, 136.5, 132.3, 129.7, 128.9, 128.5, 127.9, 126.4, 125.6, 124.9, 124.3, 113.6, 113.1, 57.1.

HRMS[M$^{+\cdot}$] $C_{17}H_{14}OS$ calculated 266.07599 found 266.0752.

Synthesis of phenyl(2,3,4,6-tetramethoxyphenyl)sulfane (3m)

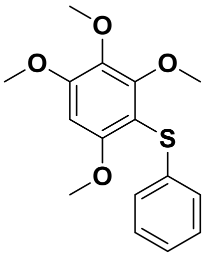

The compound was prepared according to the general procedure using 19.8 mg of phenyl(2,3,4,6-tetramethoxyphenyl)sulfane, 2.2 mg of $[Ir(dF(CF_3)ppy)_2(dtbpy)]PF_6$, 43.6 mg of diphenyl disulfide, 40 mg of $(NH_4)_2S_2O_8$ and 2.0 mL dry CH_3CN. The reaction mixture was irradiated for 24 hours under N_2. Purification of the crude product was achieved by flash column chromatography using petrol ether/ ethylacetate (85:15) as eluent to yield the desired compound as white solid (12.2 mg, 40%).

^{1}H NMR ($CDCl_3$, 300 MHz, ppm): δ 7.199-7.149 (m, 2H), 7.072-7.021 (m, 3H), 6.375 (s, 1H), 3.936 (s, 3H), 3.831 (s, 3H), 3.801 (s, 3H), 3.796 (s, 3H).

^{13}C NMR ($CDCl_3$, 75 MHz, ppm): δ 157.7, 156.5, 155.6, 138.9, 137.2, 128.8, 126.2, 124.9, 92.9, 61.8, 61.4, 56.8, 56.2.

HRMS[M$^{+\cdot}$] $C_{16}H_{18}O_4S$ calculated 306.0920 found 306.0922.

Crystallographic Data

Single crystals of **3d**, **3e** and **3i** were obtained by recrystallisation from DCM/PE and Single crystals of **3a** was obtained by recrystallisation from EA/PE.

**Compound**	**3a**	**3d**	**3e**	**3i**
CCDC No	1847083	1847084	1847085	1847086
Formula	$C_{21}H_{20}O_3S_2$	$C_{12}H_{17}NO_3S_2$	$C_{20}H_{18}O_2S_2$	$C_{12}H_{17}NO_3S_2$
$D_{calc.}$/ g cm^{-3}	1.324	1.420	1.363	1.343
μ/mm^{-1}	2.645	3.604	2.863	3.410
Formula Weight	384.49	287.38	354.46	287.38
Colour	clear colourless	clear colourless	clear colourless	Clear colourless
Shape	plate	block	plate	plate
Max Size/mm	0.28	0.12	0.19	0.13
Mid Size/mm	0.17	0.10	0.14	0.08
Min Size/mm	0.07	0.08	0.06	0.02
T/K	122.99(10)	123.01(11)	122.9(2)	122.9(2)
Crystal System	monoclinic	monoclinic	monoclinic	triclinic
Space Group	$P2_1/c$	$P2_1/n$	$P2_1/c$	P-1
a/Å	14.5868(3)	7.51380(10)	10.3054(4)	7.3374(3)
b/Å	17.6123(3)	7.37880(10)	11.0809(4)	10.3449(5)
c/Å	7.5316(2)	24.3461(4)	15.6403(5)	10.3830(8)
α/°	90	90	90	74.208(6)
β/°	94.518(2)	95.064(2)	104.792(4)	75.907(5)
γ/°	90	90	90	72.065(4)
V/Å^3	1928.91(7)	1344.55(3)	1726.82(11)	710.46(8)
Z	4	4	4	2
Z'	1	1	1	1
Wavelength/Å	1.54184	1.54184	1.54184	1.54184
Radiation type	CuK$_\alpha$	CuK$_\alpha$	CuK$_\alpha$	CuK$_\alpha$
Θ_{min}/°	3.942	3.645	4.437	4.494
Θ_{max}/°	73.926	73.882	74.823	75.226
Measured Refl.	40564	17615	24757	2858
Independent Refl.	3891	2702	3513	2858
Reflections Used	3496	2472	3070	2321
R_{int}	0.0555	0.0299	0.0465	0.0469
Parameters	238	168	219	169
Restraints	0	0	0	0
Largest Peak	0.259	0.321	0.242	0.564
Deepest Hole	-0.232	-0.303	-0.325	-0.392
GooF	1.032	1.042	1.050	1.090
wR_2 (all data)	0.0759	0.0703	0.0798	0.1515
wR_2	0.0718	0.0676	0.0763	0.1391
R_1 (all data)	0.0344	0.0297	0.0372	0.0749
R_1	0.0296	0.0263	0.0306	0.0614

^{1}H- and ^{13}C NMR spectra of synthesized compounds

^{1}H spectra of compound 3a ($CDCl_3$, 300 MHz)

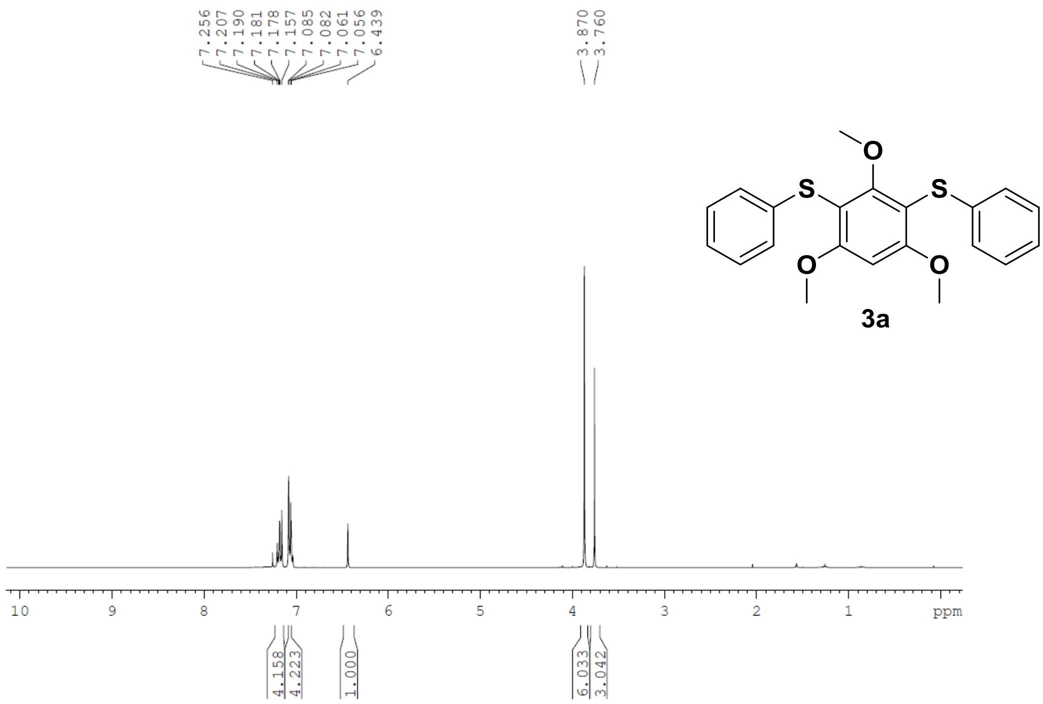

^{13}C spectra of compound 3a ($CDCl_3$, 75 MHz)

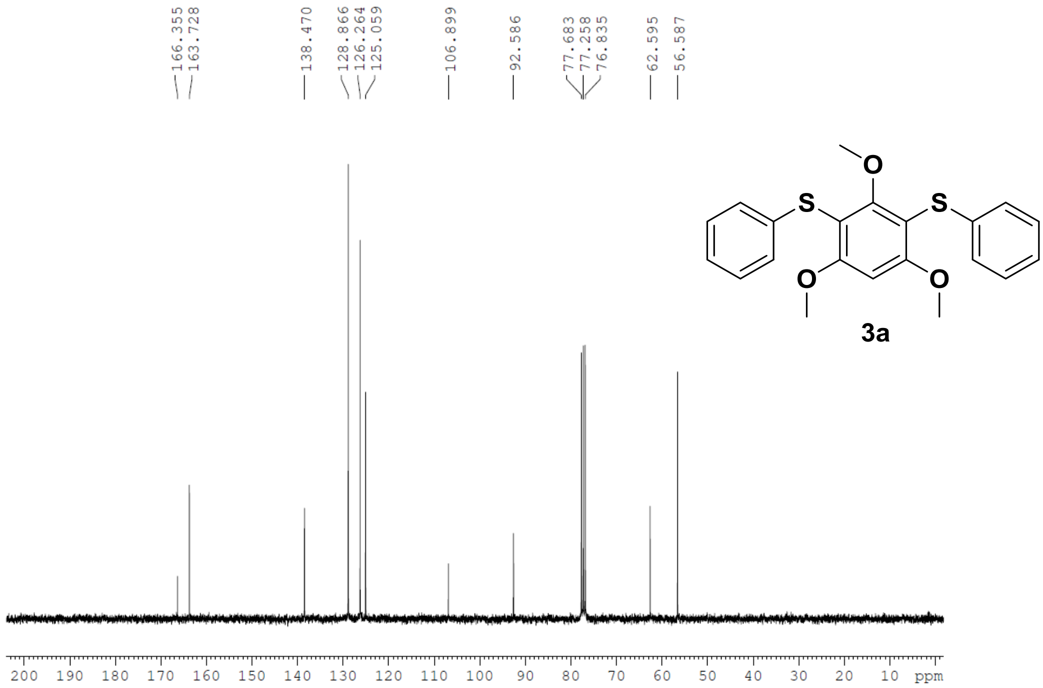

^{1}H spectra of compound 3b ($CDCl_3$, 300 MHz)

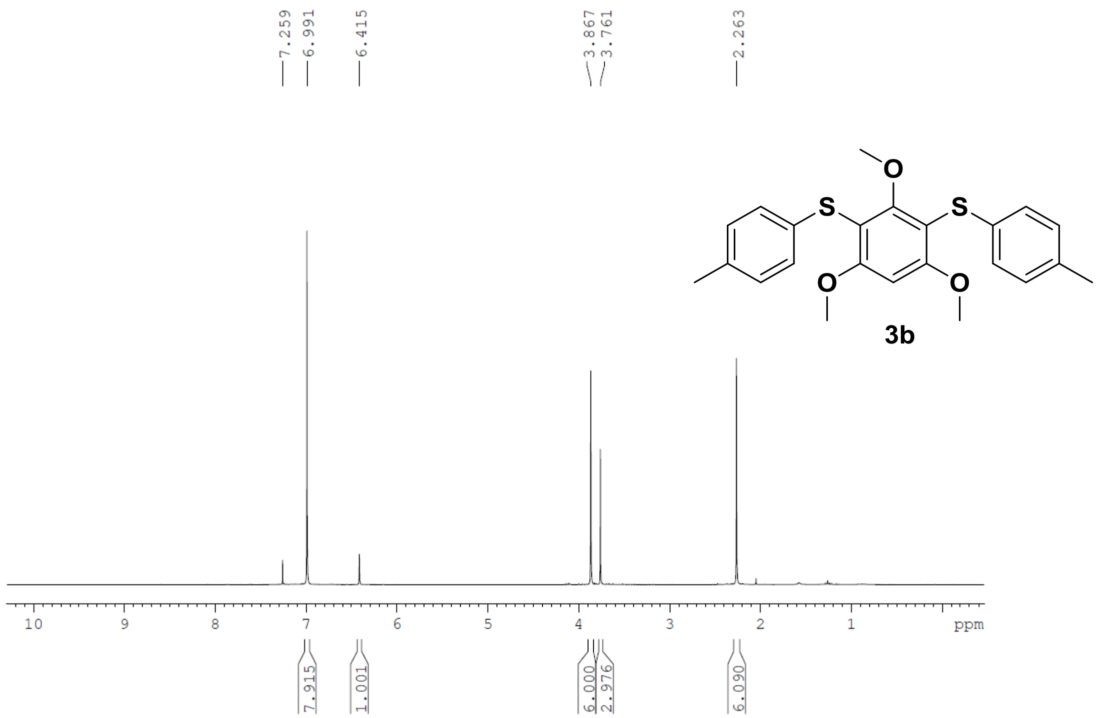

^{13}C spectra of compound 3b ($CDCl_3$, 75 MHz)

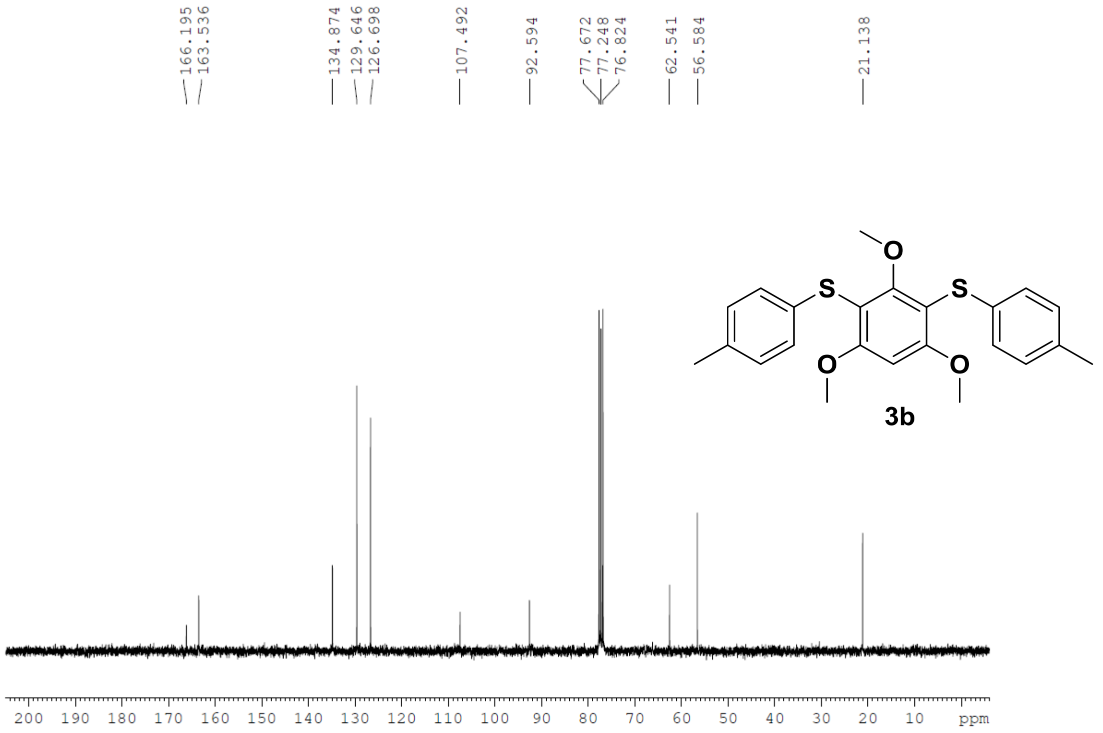

^{1}H spectra of compound 3c ($CDCl_3$, 300 MHz)

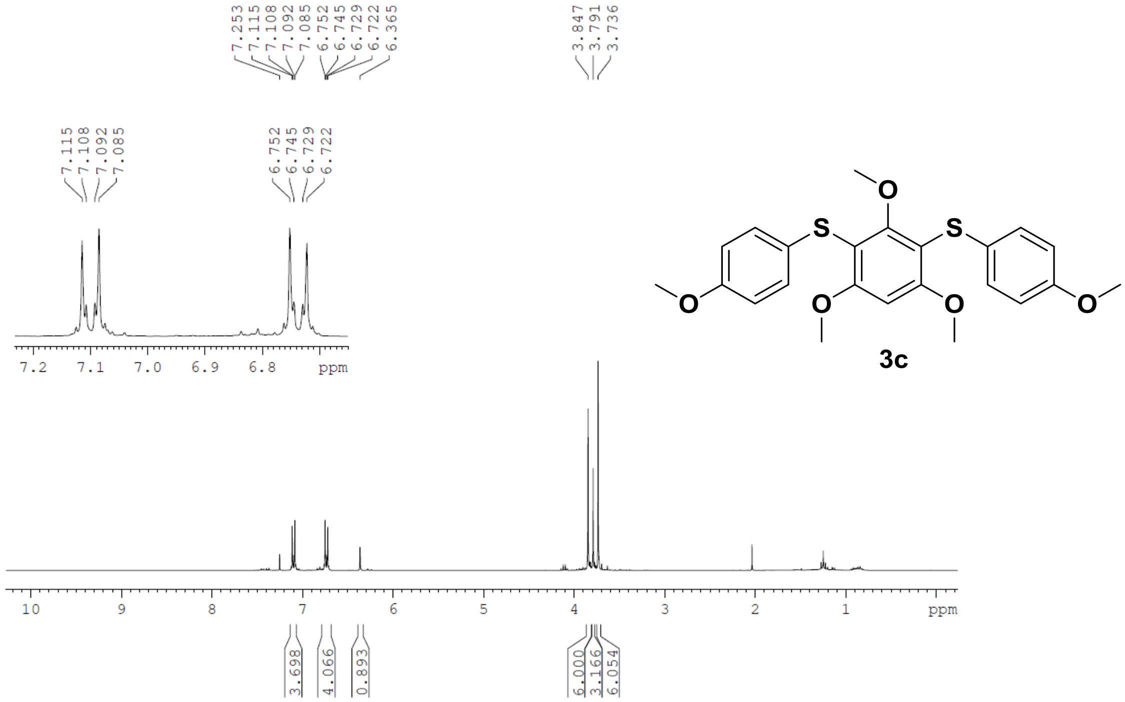

3c

^{13}C spectra of compound 3c ($CDCl_3$, 75 MHz)

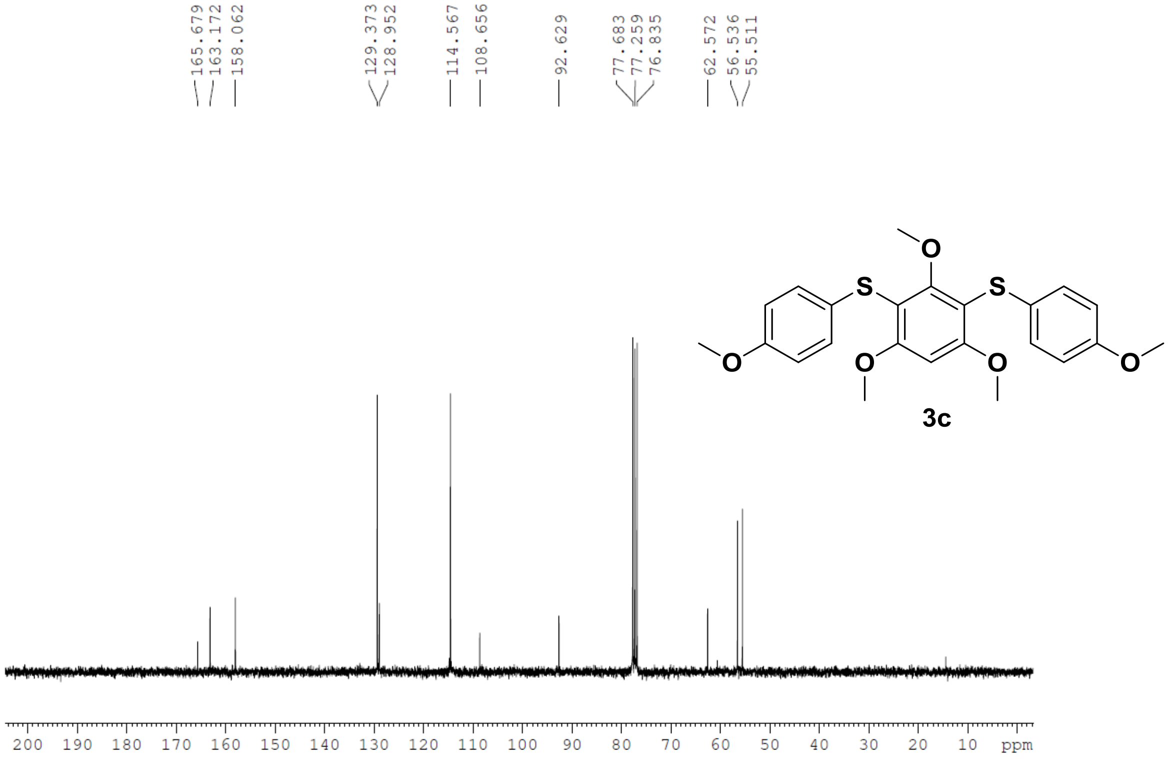

3c

^{1}H spectra of compound 3d ($CDCl_3$, 300 MHz)

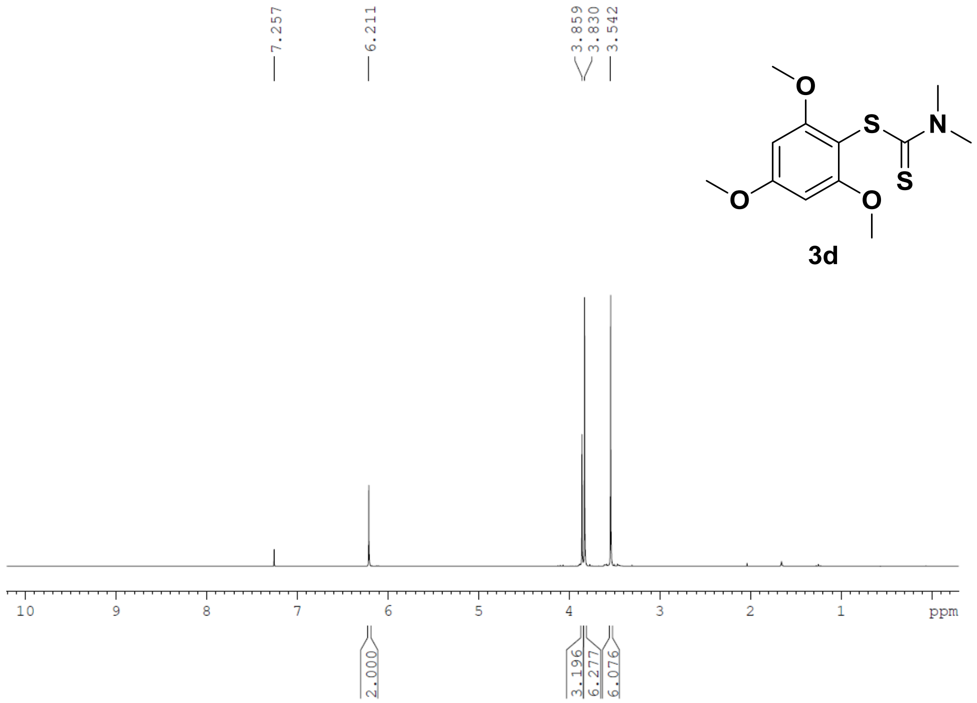

^{13}C spectra of compound 3d ($CDCl_3$, 75 MHz)

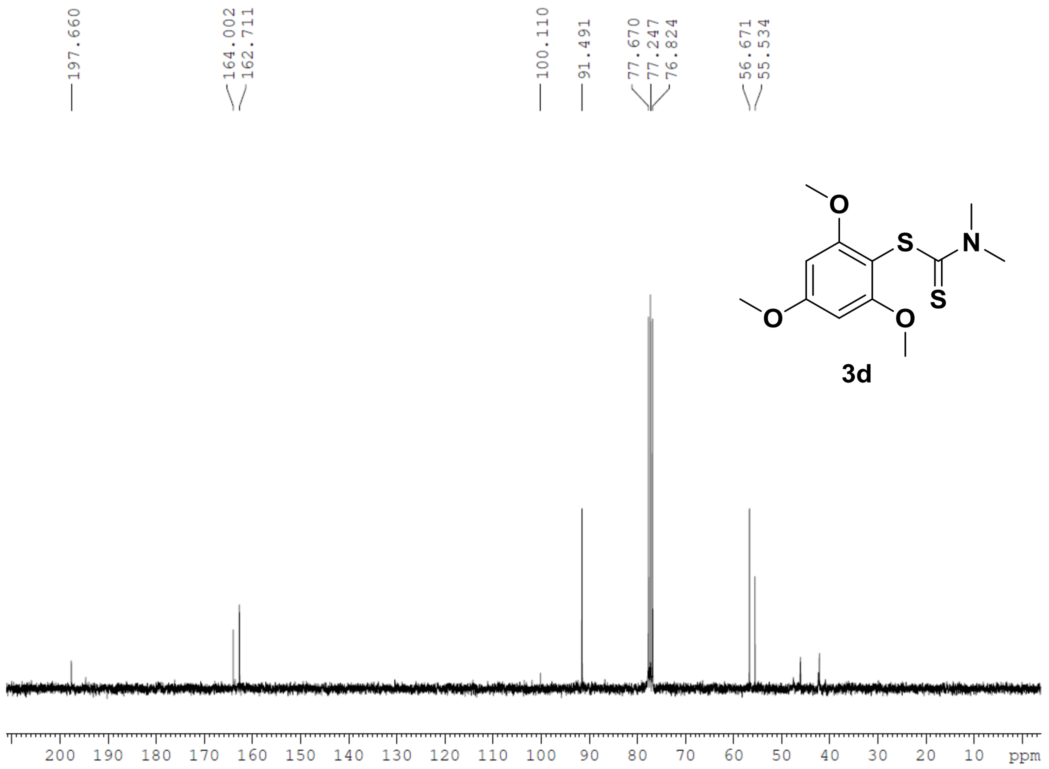

^{1}H spectra of compound 3e ($CDCl_3$, 300 MHz)

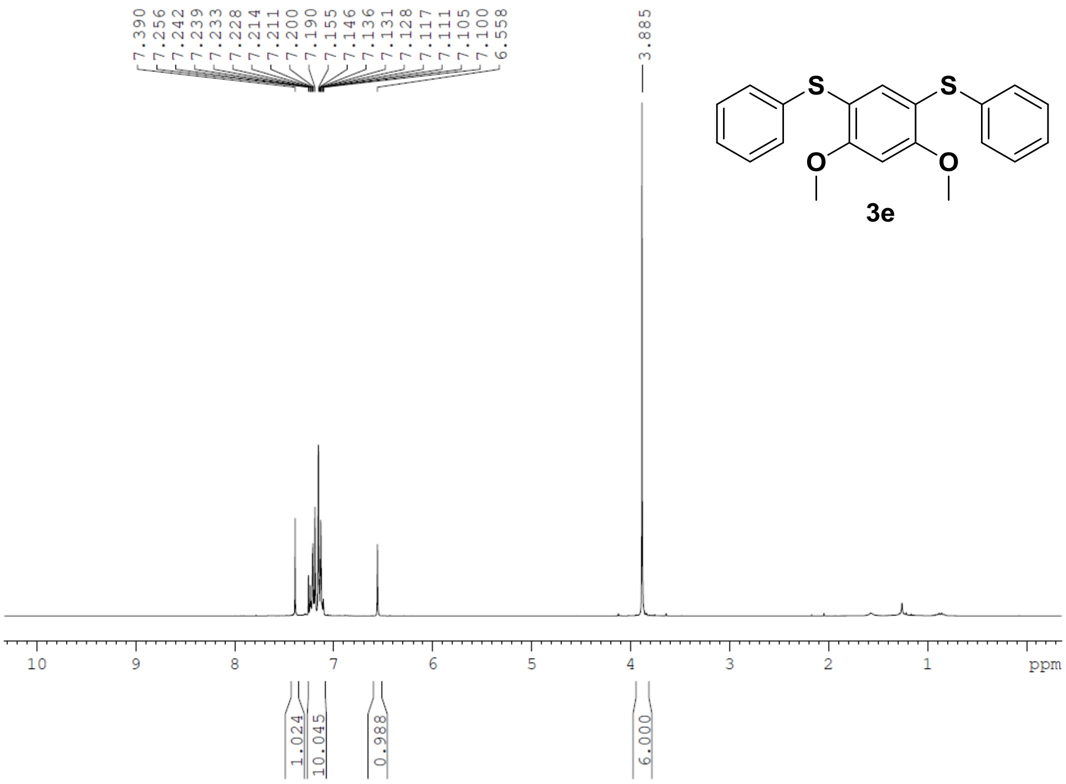

^{13}C spectra of compound 3e ($CDCl_3$, 75 MHz)

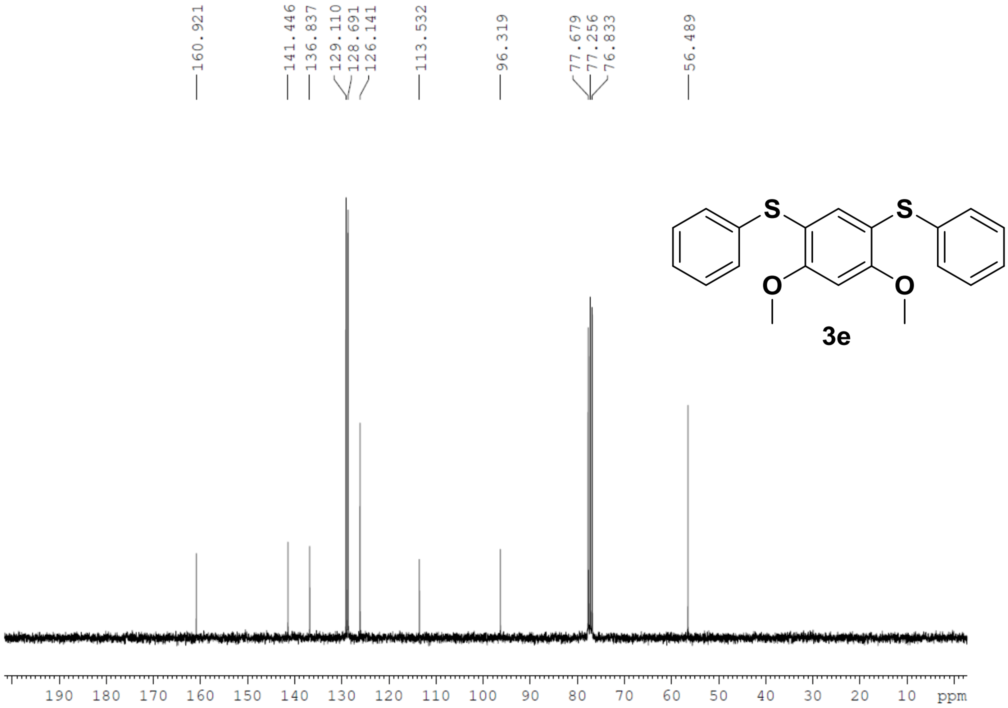

^{1}H spectra of compound 3f ($CDCl_3$, 300 MHz)

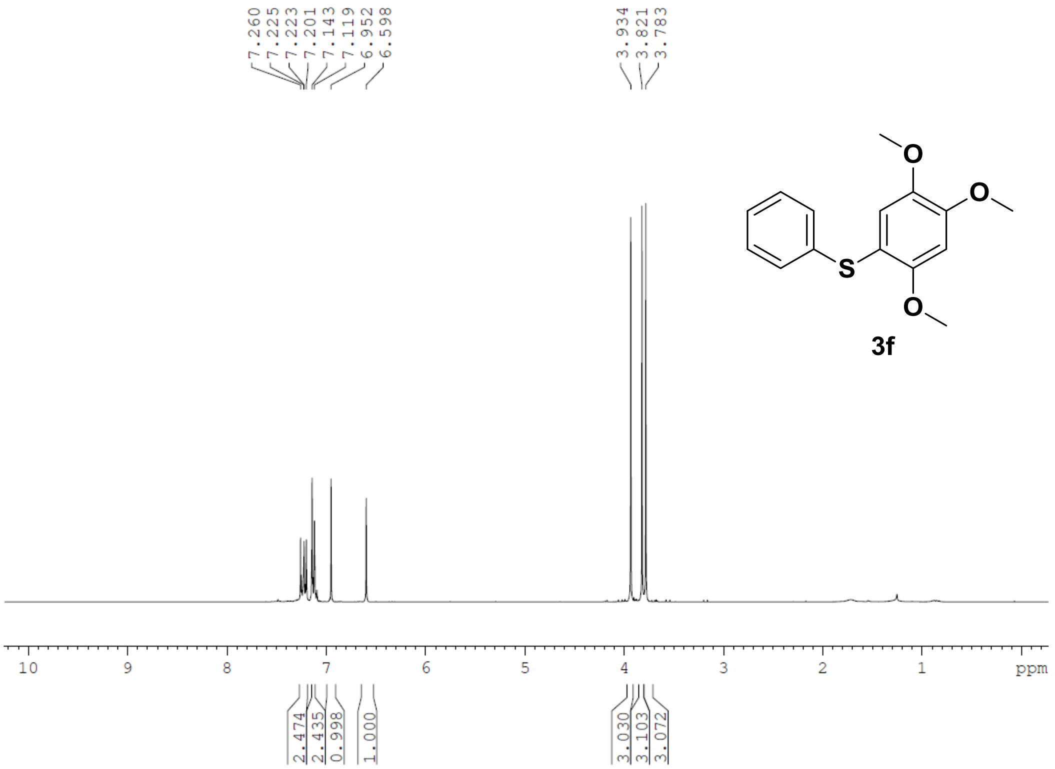

^{13}C spectra of compound 3f ($CDCl_3$, 75 MHz)

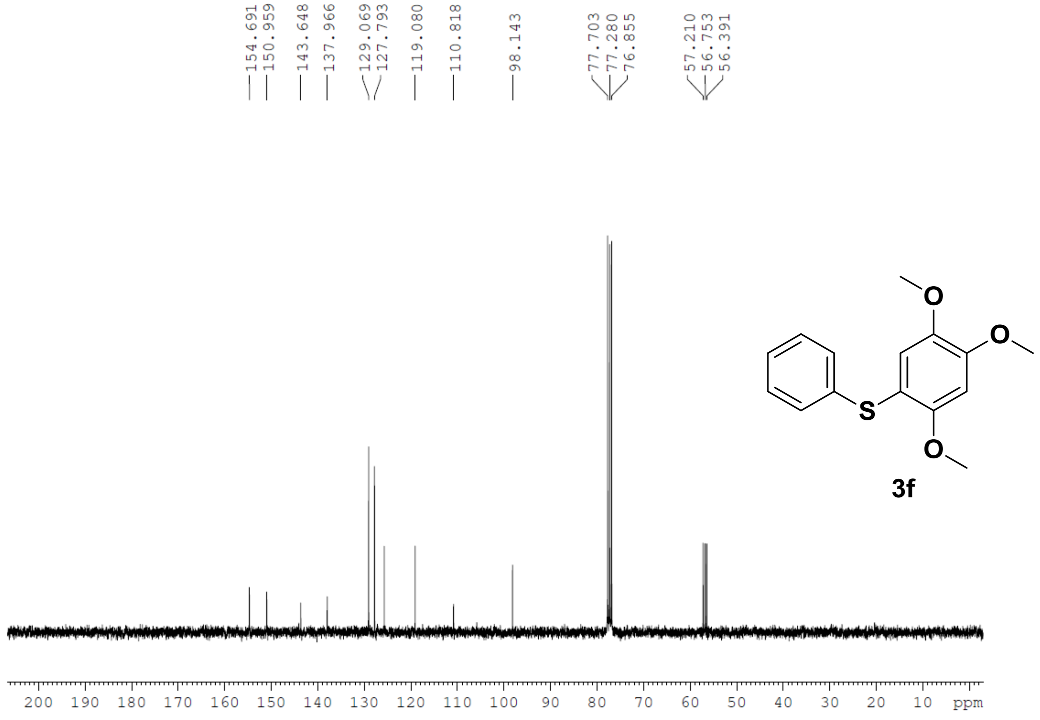

^{1}H spectra of compound 3g ($CDCl_3$, 300 MHz)

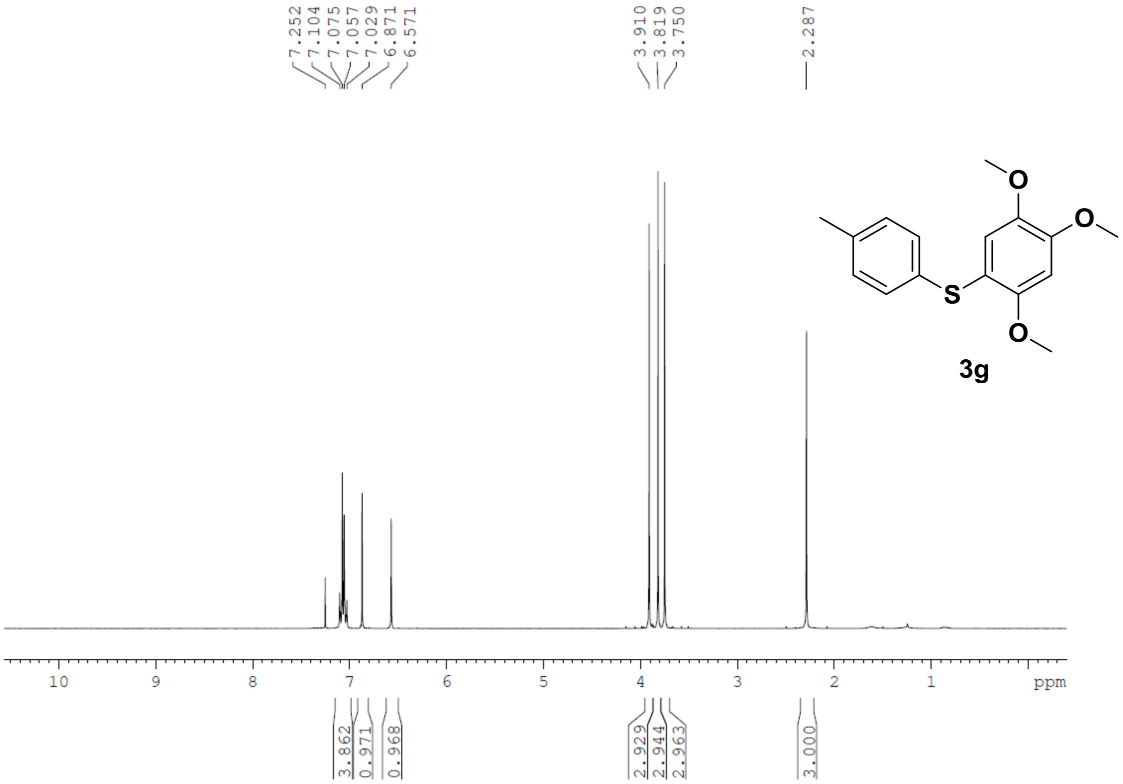

^{13}C spectra of compound 3g ($CDCl_3$, 75 MHz)

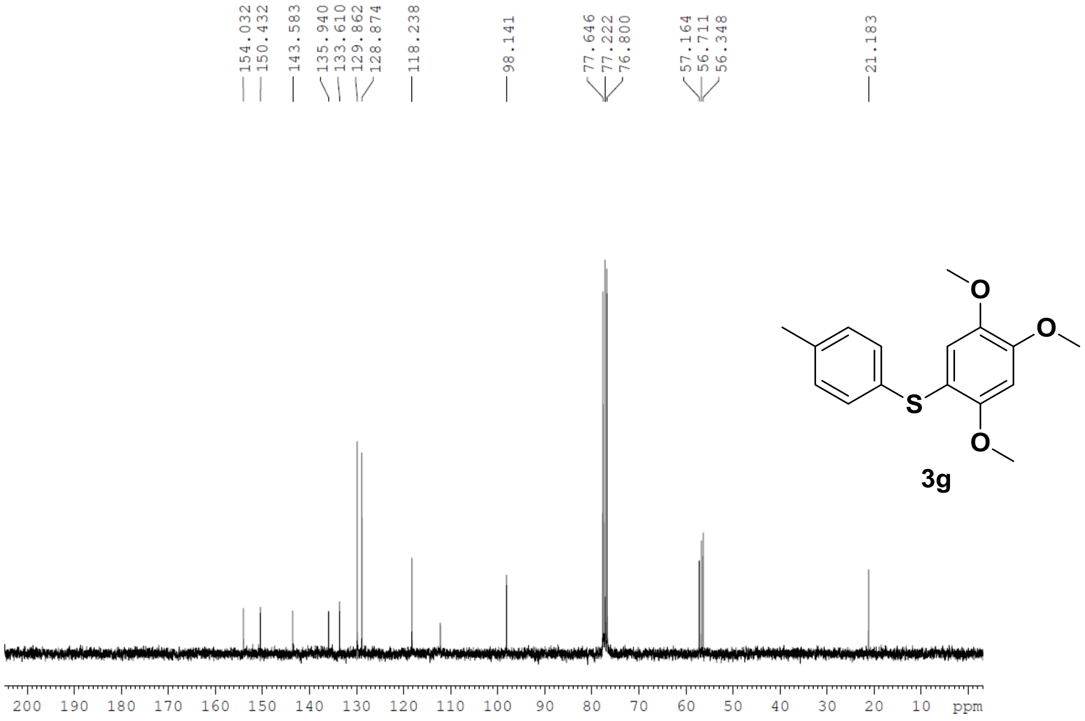

^{1}H spectra of compound 3h ($CDCl_3$, 300 MHz)

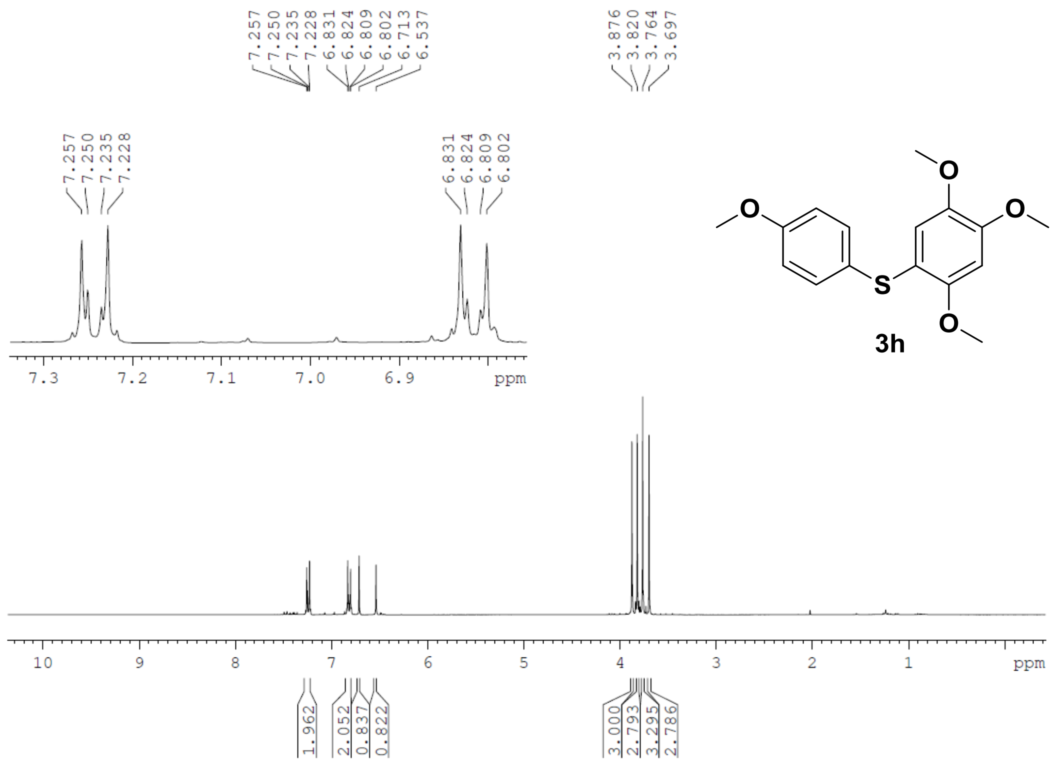

^{13}C spectra of compound 3h ($CDCl_3$, 75 MHz)

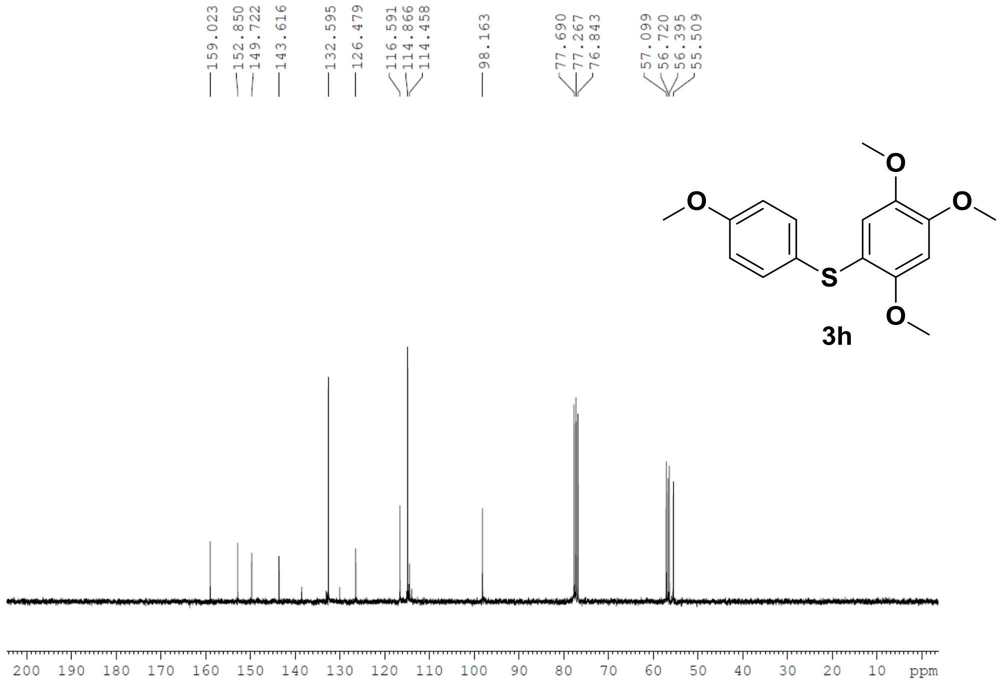

^{1}H spectra of compound 3i ($CDCl_3$, 300 MHz)

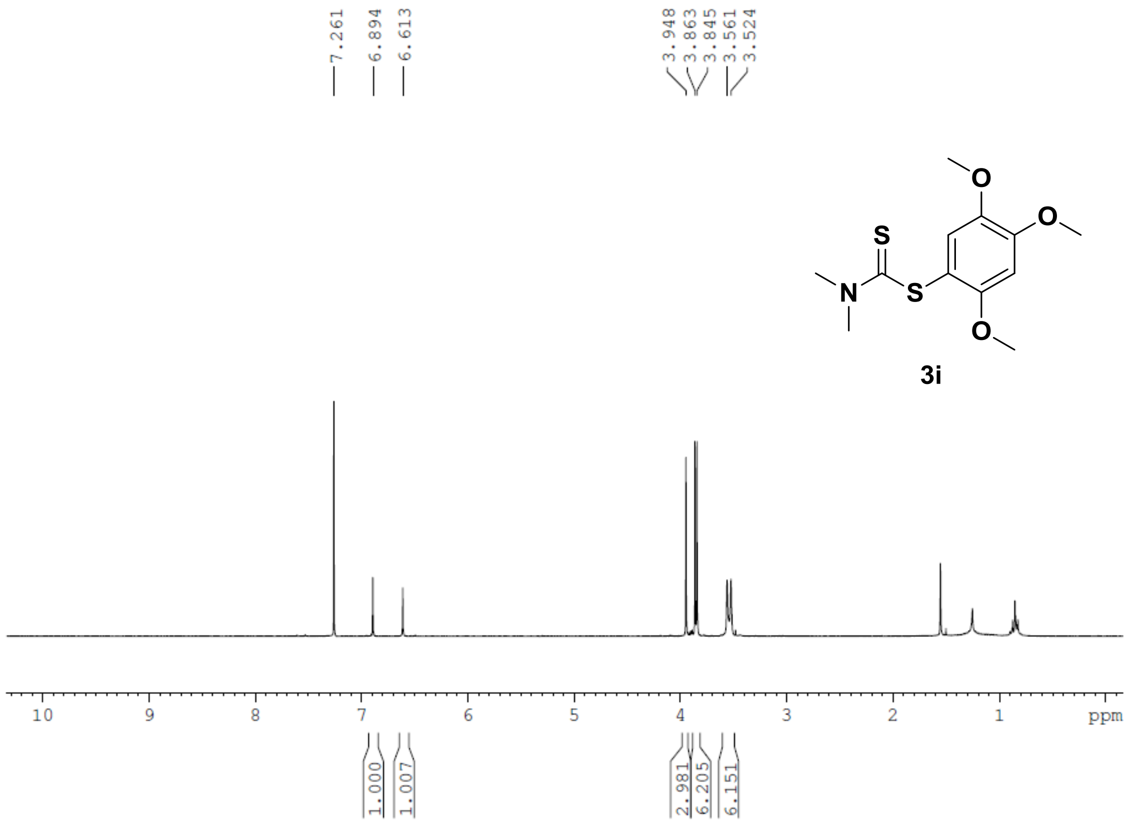

^{13}C spectra of compound 3i ($CDCl_3$, 75 MHz)

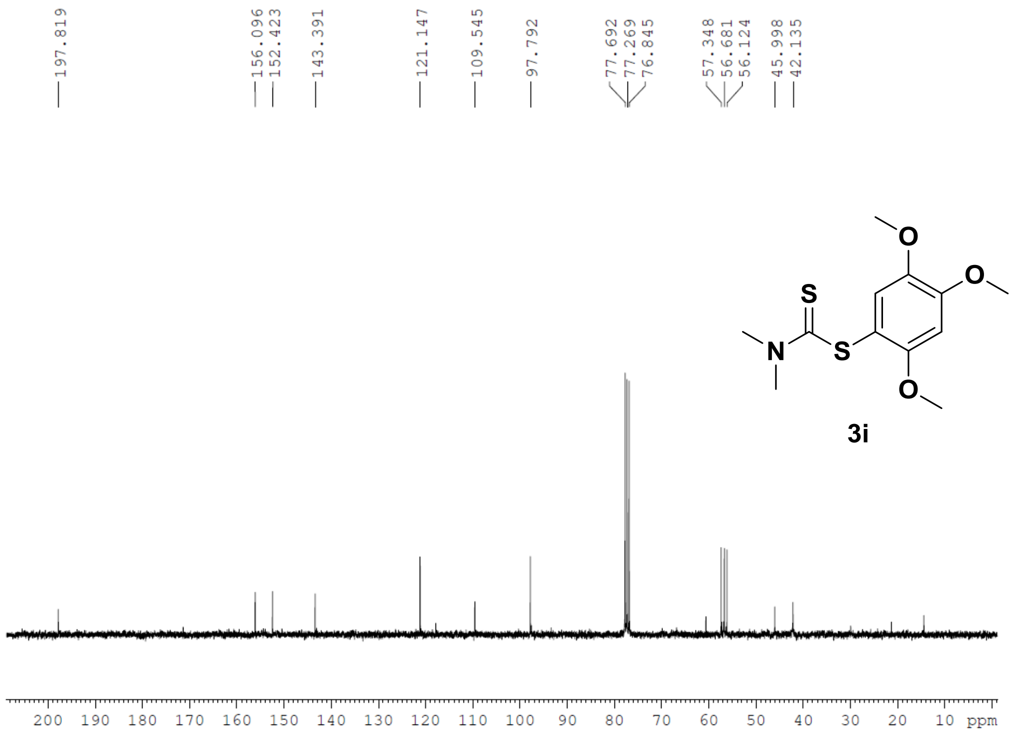

^{1}H spectra of compound 3j ($CDCl_3$, 600 MHz)

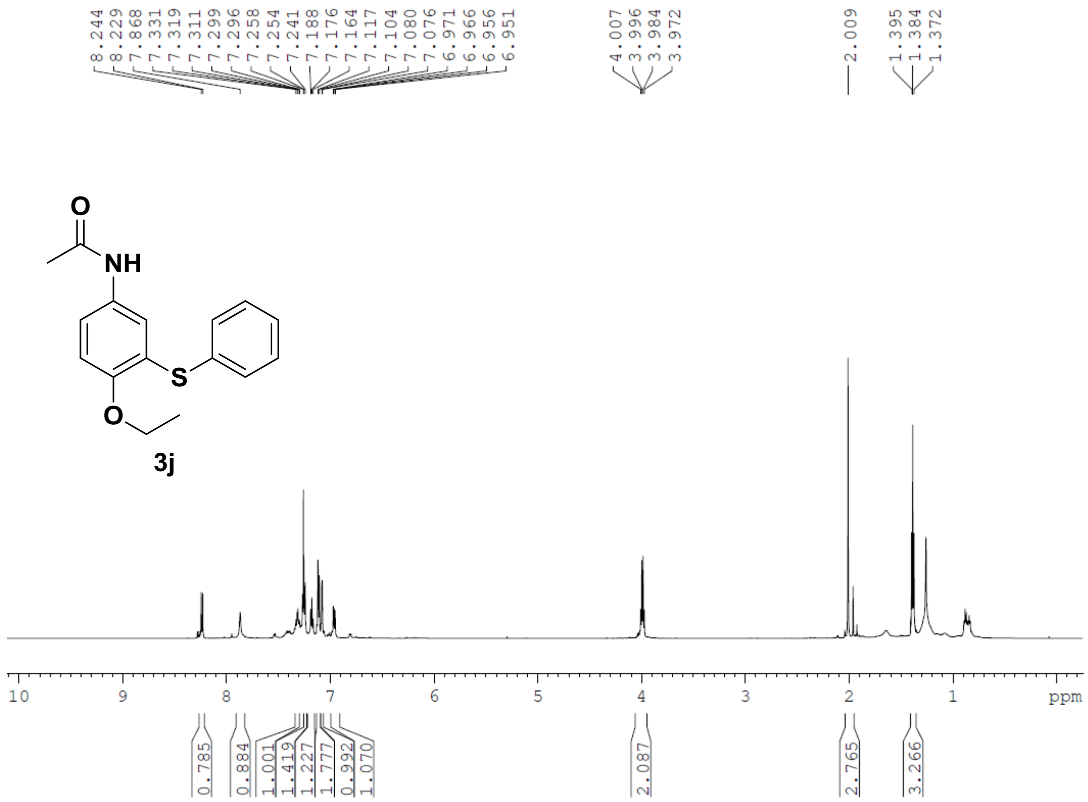

^{13}C spectra of compound 3j ($CDCl_3$, 151 MHz)

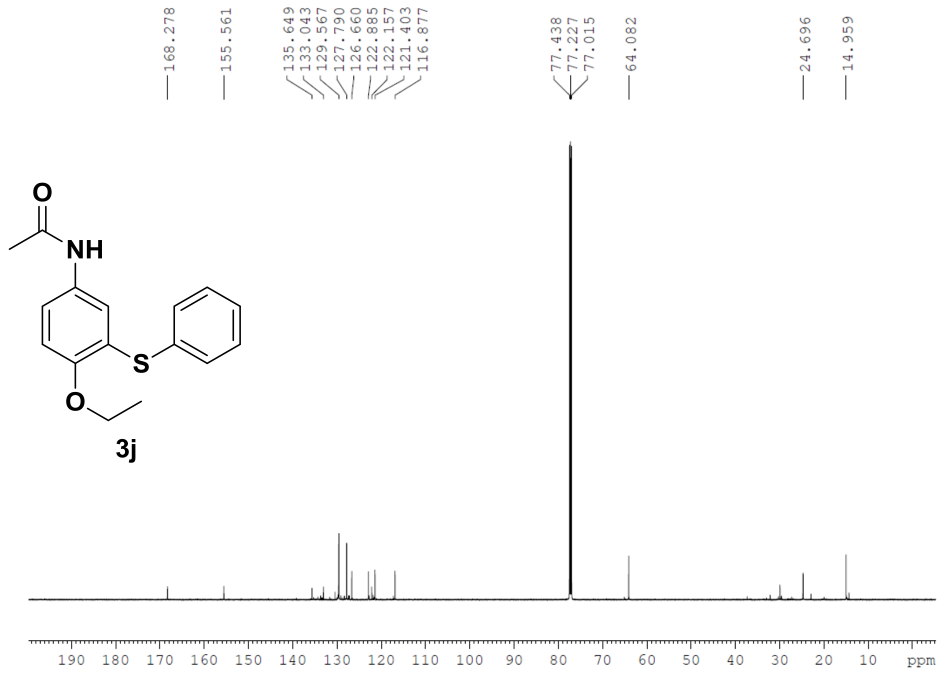

^{1}H spectra of compound 3k ($CDCl_3$, 600 MHz)

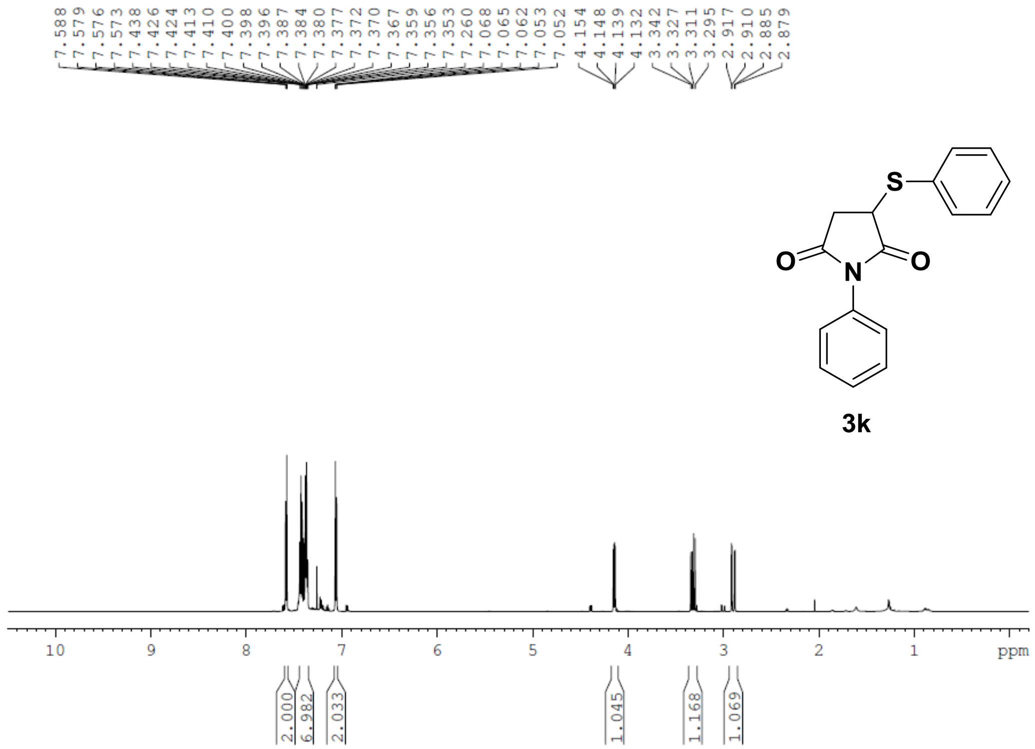

^{13}C spectra of compound 3k ($CDCl_3$, 151 MHz)

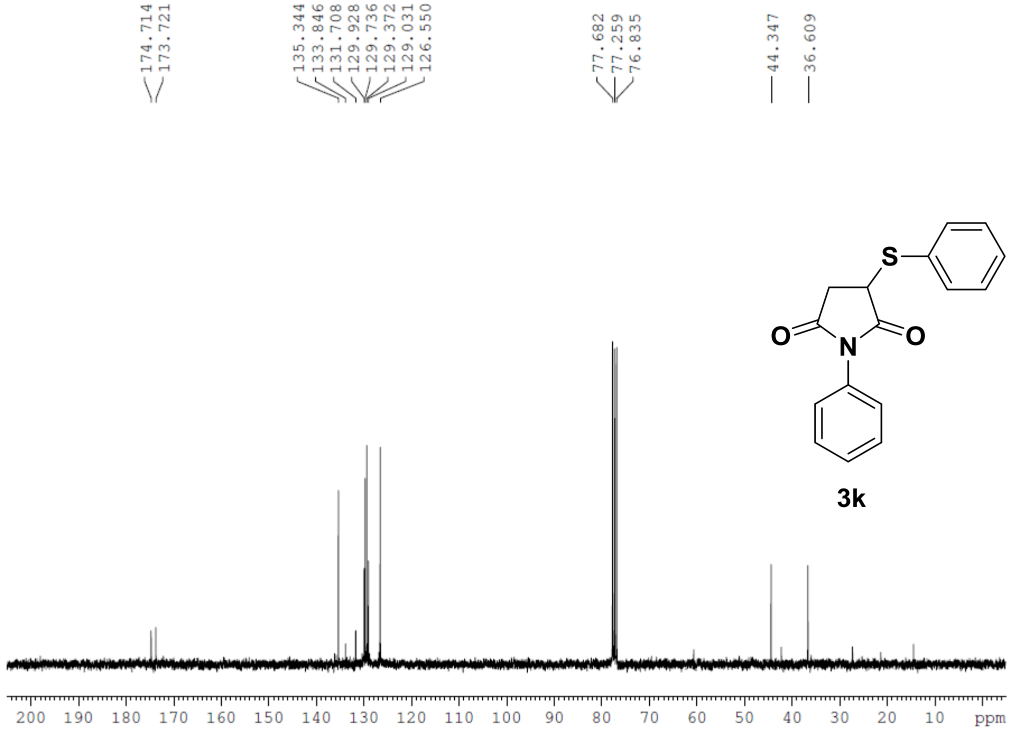

^{1}H spectra of compound 3l ($CDCl_3$, 300 MHz)

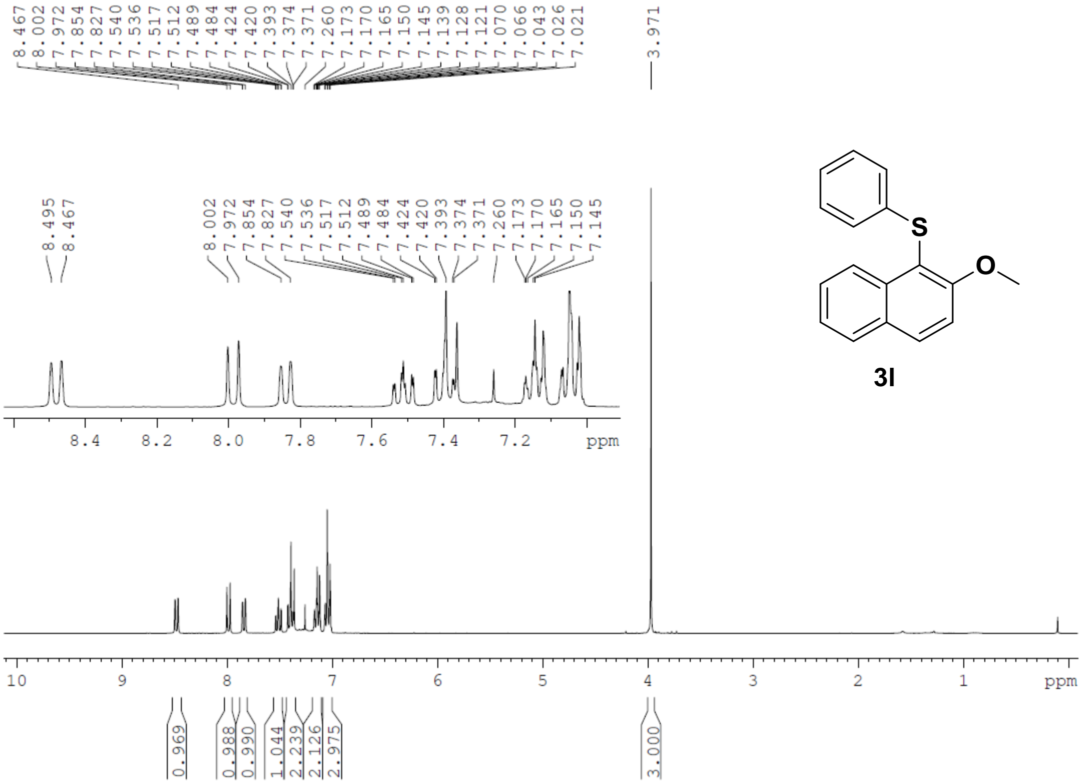

^{13}C spectra of compound 3l ($CDCl_3$, 75 MHz)

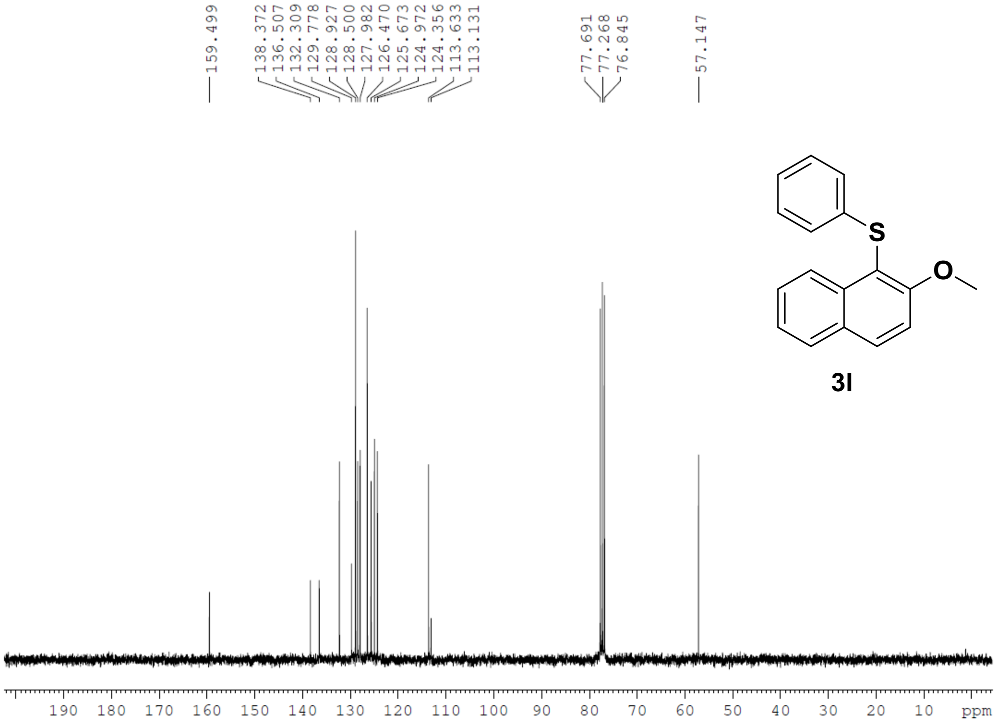

^{1}H spectra of compound 3m ($CDCl_3$, 300 MHz)

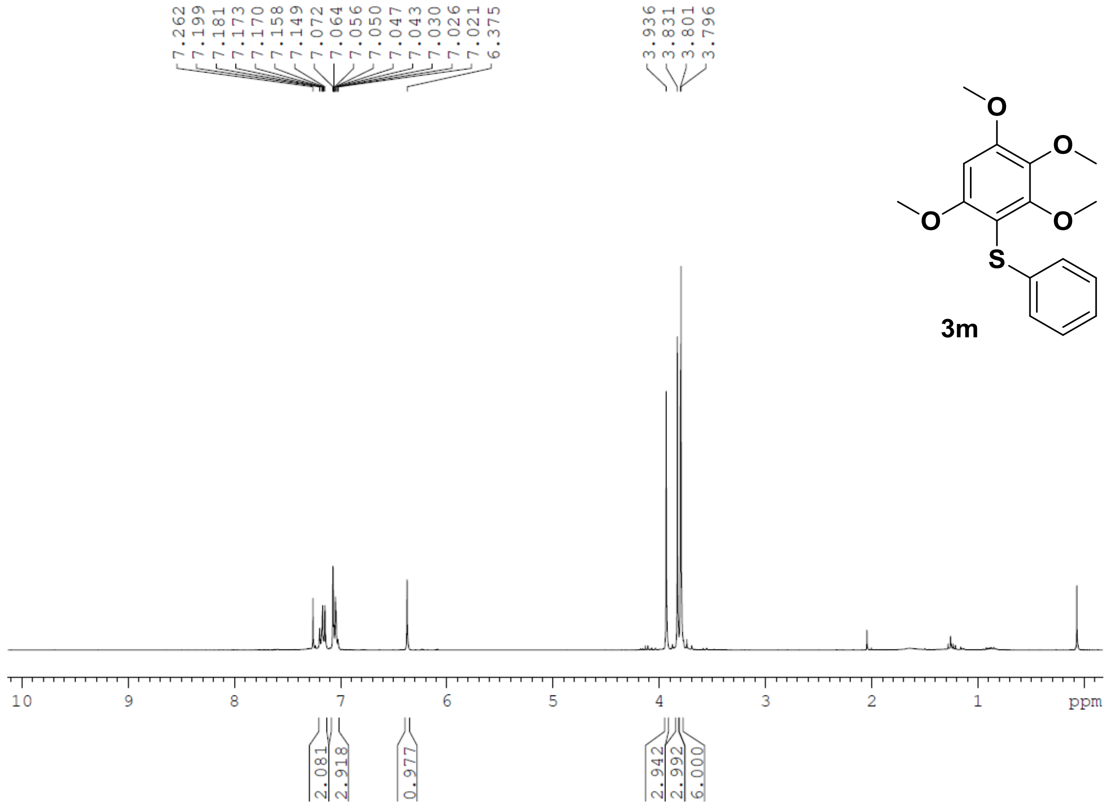

^{13}C spectra of compound 3m ($CDCl_3$, 75 MHz)

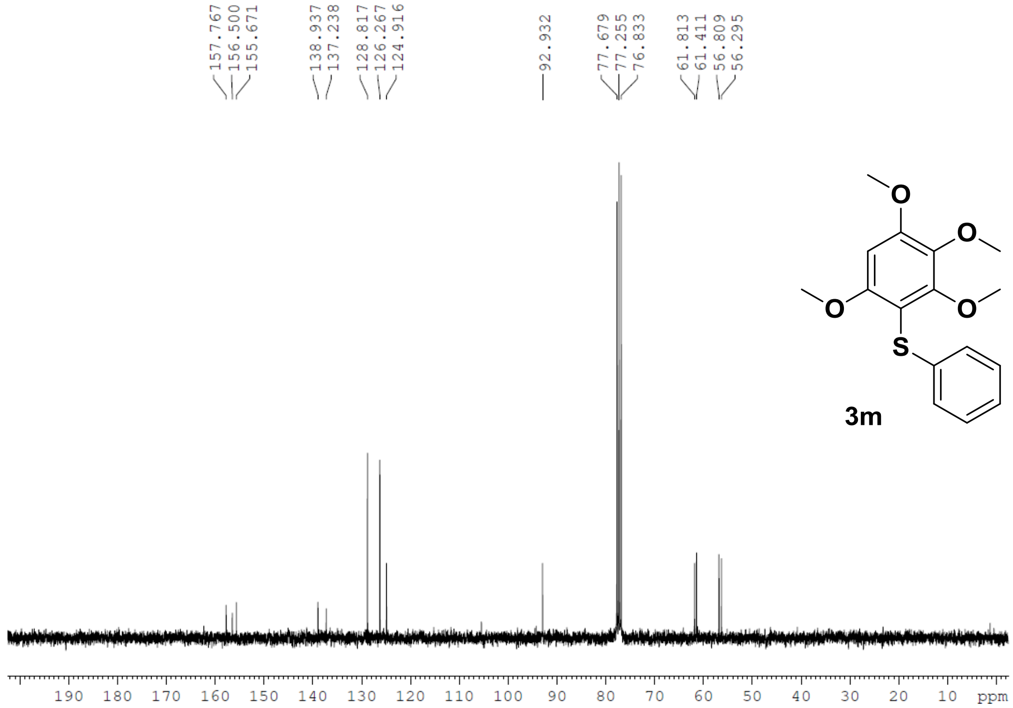

References

1. D. B. G. Williams, M. J. Lawton, *J. Org. Chem*. **2010**, *75*, 8351-8354.
2. M. S. Lowry, J. I. Goldsmith, J. D. Slinker, R. Rohl, R. A. Pascal, G. G. Malliaras, *Chem. Mater*. **2005**, *17*, 5712−5719.
3. O. V. Dolomanov, L. J. Bourhis, R. J. Gildea, J. A. K. Howard, H. Puschmann, *J. Appl. Crystallogr*. **2009**, *42*, 339-341.
4. G. M. Sheldrick, *Acta Crystallogr. C*, **2015**, *71*, 3-8.
5. L. Chen, P. Liu, J. Wu, B. Dai, *Tetrahedron.* **2018**, *74*, 1513-1519.
6. K. Yan, D. Yang, P. Sun, W. Wei, Y. Liu, G. Li, S. Lu, H. Wang, *Tetrahedron. Lett*. **2015**, *56,* 4792-4795.
7. M. R. Reddy, G. S Kumar, H. M. Meshram, *Tetrahedron. Lett*. **2016**, *57,* 3622-3624.
